# Impact of mobile connectivity on students’ wellbeing: Detecting learners’ depression using machine learning algorithms

**DOI:** 10.1371/journal.pone.0294803

**Published:** 2023-11-27

**Authors:** Muntequa Imtiaz Siraji, Ahnaf Akif Rahman, Mirza Muntasir Nishat, Md Abdullah Al Mamun, Fahim Faisal, Lamim Ibtisam Khalid, Ashik Ahmed

**Affiliations:** 1 Department of Electrical and Electronic Engineering, Islamic University of Technology, Gazipur, Dhaka, Bangladesh; 2 Department of Technical and Vocational Education, Islamic University of Technology, Gazipur, Dhaka, Bangladesh; Universiti Tunku Abdul Rahman, MALAYSIA

## Abstract

Depression is a psychological state of mind that often influences a person in an unfavorable manner. While it can occur in people of all ages, students are especially vulnerable to it throughout their academic careers. Beginning in 2020, the COVID-19 epidemic caused major problems in people’s lives by driving them into quarantine and forcing them to be connected continually with mobile devices, such that mobile connectivity became the new norm during the pandemic and beyond. This situation is further accelerated for students as universities move towards a blended learning mode. In these circumstances, monitoring student mental health in terms of mobile and Internet connectivity is crucial for their wellbeing. This study focuses on students attending an International University of Bangladesh to investigate their mental health due to their continual use of mobile devices (e.g., smartphones, tablets, laptops etc.). A cross-sectional survey method was employed to collect data from 444 participants. Following the exploratory data analysis, eight machine learning (ML) algorithms were used to develop an automated normal-to-extreme severe depression identification and classification system. When the automated detection was incorporated with feature selection such as Chi-square test and Recursive Feature Elimination (RFE), about 3 to 5% increase in accuracy was observed by the method. Similarly, a 5 to 15% increase in accuracy has been observed when a feature extraction method such as Principal Component Analysis (PCA) was performed. Also, the SparsePCA feature extraction technique in combination with the CatBoost classifier showed the best results in terms of accuracy, F1-score, and ROC-AUC. The data analysis revealed no sign of depression in about 44% of the total participants. About 25% of students showed mild-to-moderate and 31% of students showed severe-to-extreme signs of depression. The results suggest that ML models, incorporating a proper feature engineering method can serve adequately in multi-stage depression detection among the students. This model might be utilized in other disciplines for detecting early signs of depression among people.

## 1 Introduction

Depression is a state of the human mind that often reaps undesirable effects on an individual’s feelings, motivations, and actions. It gradually inhibits the day-to-day activities and attention of a person and leads to a general loss of pleasure and interest in work. This state of mind often serves as a contributing factor for many psychological and physical complications such as diabetes, heart disease etc. [1, 2]. Approximately 3.8% of the total population worldwide undergoes this sort of mental disorder [3]. In Bangladesh, 6.4 million people (4.10%) experience different depressive disorders, and more than 75% of people in such Low to Middle-Income Countries (LMICs) fail to receive proper care for their mental health [4, 5]. In addition, mistreating depression can instigate self-destructing tendencies and often lead to the most common psychiatric illnesses linked to abnormal death [6–8]. A biochemical or neurophysiological response (e.g., Monoamine turnover—noradrenaline, serotonin, or 5-HT) is also assumed to relate to depression in the individuals [9].

Since the beginning of 2020, the Covid-19 pandemic caused a paradigm shift to people’s lives and introduced unprecedented changes to their usual lifestyles. For example, all in-person activities were suspended across the educational institutions substituted by an online education system that was introduced within a brief time. The hands-on experience was replaced with the continuous use of digital tools and learning over the internet via mobile screens. Research reported that initially the students were struggling to cope with the situation [10], but by the end of that year had become habituated to the remote learning system [11].

Although the hybrid mode of learning was found to be promising in the event of a disruption in academic processes [12], its applicability needs to be tested for different populations. LMICs such as Bangladesh lack the facilities of a robust infrastructure such as a stable electricity grid, adequate internet connections, sufficient on-campus resources, technical knowledge, and sufficient competency of teachers to support the students. Multiple reports showed that students were negatively impacted by these issues.

Under this new learning context, monitoring a student’s mental health is essential to the process of providing a favorable learning environment for promoting well-being. Although depression can be identified among all age groups, students are particularly susceptible to it as the new education delivery and management system affects them the most. A student can experience depressive episodes of single, recurrent, or bipolar nature [13] which can greatly affect their psychological well-being as well as social-work life. Students pursuing their undergraduate degrees need to coordinate a lot of tasks and responsibilities such as regular coursework, lab assignments, optional projects, etc., which can become cumbersome and depressing at times [14]. Thus, university authorities should be aware of student mental health in this new learning environment.

Previous studies show that without proper diagnosis, care, and treatment, a student with mental depression can face severe challenges to cope with their learning [15]. However, clinical assessment of mass undergraduate students would be unwieldy and resource-heavy, since there can be numerous factors involved in instigating depression. In this regard, Machine Learning (ML) models [16–25] can perhaps become valuable for detecting and predicting subsequent health issues [26–40] as well as depressive episodes. Furthermore, the result can be analyzed to identify depression-related trends revealed among young people which can aid higher education institutions to understand the factors better and develop effective strategies to mitigate these factors.

Depression, anxiety, and stress scale (DASS-21) is a popular tool to measure students’ depression. Numerous studies have been performed to assess the validity and reliability of this tool [41–44]. Javaeed et al. found a mild positive correlation between internet addiction and depression when employing the DASS-21 scale in undergraduate medical students of Kashmir [45]. Prediction of depression was accomplished by Priya et al. who employed five different machine learning algorithms on the data collected using DASS-21 scale while the Naïve Bayes algorithm achieved the best accuracy [46]. A similar prediction of anxiety, depression and stress using DASS-42 scale was also revealed by Kumar et al. in five severity levels using eight machine learning algorithms [47]. Oei et al. proposed a revised DASS-18 scale for the Asian population to overcome any cultural influence [48].

Mutalib et al. performed a similar analysis on higher education students in Malaysia by utilizing the World Health Organization Quality of Life (WHOQOL) tool to identify factors that are responsible for mental health problems [49]. Elhai et al. researched problematic smartphone use (PSU) of Chinese undergraduate students to identify the severity of mental health and its correlation with psychopathology variables [50]. Muzammel et al. investigated the effect of phoneme vowels and consonants for recognizing clinical depression from speech [51]. Yasin et al. made a review study on EEG-based major and bipolar depressive disorder detection where the state-of-the-art neural networks were thoroughly discussed [52]. Othmani et al. performed several studies on depression detection from speech and audio-visual data [53–55]. They explored a Model of Normality in producing results more efficiently by examining the audio-visual patterns of a depressed subject to that of a symptom-free one. They also proposed EmoAudioNet for both emotion and major depressive disorder detection and a correlation-based network where depression relapse is detected. Richter et al. [56, 57] designed two studies using a machine learning approach to identify the differences in behavioral patterns stemming from anxiety and depression disorder.

In the context of Bangladesh, Islam et al. [58] performed a statistical analysis of adolescent mental health by employing g a Patient Health Questionnaire (PHQ-9) and Generalized Anxiety Disorder Assessment (GAD-7) scale to ascertain mental health conditions prevalent among the students. Ahmmed et al. [59] attempted to identify the effect of the global pandemic on the psychological well-being and academic studies of undergraduate students. Using ML algorithms, Ahnaf et al. [60] studied a sample of 577 undergraduate students to determine the reasons for the frequent occurrence of depression in this cohort. Munir et al. [61] tried to address the inadequate information regarding depression predictors using ML classifiers. Nayan et al. [62] intended to differentiate between anxiety and depression during the first wave of coronavirus among university students and achieved high accuracy (91.49%) of detection with Support Vector Machine classifier [63]. One of the notable contributions came from Zulfiker et al. [63] who employed six machine learning classifiers using various socio-demographic variables and psychosocial information to predict depression among the students. They used different feature selection methods and Synthetic Minority Oversampling Technique (SMOTE) for extracting relevant features of the ML models.

The current study aimed to investigate the students’ digital well-being when the third variant of coronavirus, Omicron was rampant. It is anecdotally hypothesized that the uncertain lockdowns that were imposed during the previous variants impacted the mental health of many students. Thus, many of them may have experienced depression and other symptoms which may partly be attributed to the hybrid mode of learning during the pandemic and beyond. Specifically, the new normal accelerates the requirements of mobile and Internet connectivity among the students in their daily academic and social lives. In response to the interaction occurring between mental well-being and new digital reality, it appears no studies have been dedicated to investigating the impact of continuous mobile connectivity on students’ mental health in the context of Bangladesh [65]. Thus, a larger study was conducted employing a survey scale to investigate depression, anxiety, stress, and mental wellbeing of the students related to their mobile and internet connectivity. This current study only reports the depression component of the larger study.

## 2 Materials and methods

### 2.1 Data acquisition

#### 2.1.1 Questionnaire design

A digital well-being questionnaire was developed for the larger study consisting of 72 items in total [64]. The survey was circulated among the students using a Google form. Participants’ voluntary consent was collected prior to the survey being conducted. There were 5 items dedicated to collecting demographic information: gender, age, residence, ethnicity/geographical location, and academic level of study. Two items were targeted to obtain data related to sleeping hours and the amount of time students were connected to the Internet. Two items inquired into student satisfaction with sleep and academic study. The depression component of the questionnaire consists of 7 items. Each of these 7 items was answered on a 4-point rating scale along a continuum from *Not at all true* (0) to *Always true* (3).

This study particularly focused on depression for which the DASS-Depression subscale had been adopted. DASS-Depression has been adopted from DASS-21 which was established by Lovibond et al. in 1995 to investigate the depression of a cohort [65]. The 7 items that were utilized for this study are: *I could not seem to experience any positive feeling at all; I found it difficult to work up the initiative to do things; I felt that I had nothing to look forward t; I felt downhearted and sad; I was unable to become enthusiastic about anything; I felt I was not worth much as a person*; and *I felt that life was meaningless*.

#### 2.1.2 Ethics statement

This study is approved by the Committee for Advanced Studies and Research (CASR), Islamic University of Technology (IUT), Bangladesh. The consent was collected from the participants at the beginning of the survey.

#### 2.1.3 DASS-Depression scale

The Depression-Anxiety-Stress Scale (DASS) is a widely used survey instrument to evaluate depression experienced by the people. There are other numerous scales for depression screening such as Beck Depression Inventory (BDI), Patient Health Questionnaire (PHQ), Hamilton Rating Scale, Geriatric Depression Scale (GDI), etc. However, DASS can be regarded as a benchmark scale to evaluate the depressive mental condition of [66]. The full scale consists of 42 items which are divided into three subscales for measuring depression, anxiety, and stress. The reduced 21-item-based DASS-21 scale also exhibits similar psychometric properties as the larger scale. As discussed earlier, this scale has been validated among different populations of the world and has a good internal consistency among the depression-related items (Cronbach alpha, *α* = 0.94) [67]. Unlike some other scales, it can differentiate mood disturbances and core depression symptoms such as dysphoria, hopelessness, self-deprecation, inertia, etc. so that this characteristic has the potential to help medical practitioners to gain a better understanding of a pupil’s mental health [67, 68]. The 7-item DASS-Depression scale is one of the three self-report subscales of the DASS-21. The scoring for each item is based on a 4-point severity scale and the sum of the 7 items’ scores is multiplied by 2 to find out the depression severity score of each participant [69]. These five depression severity levels are determined as follows:

- Normal (scores 0–9)–labelled as 0
- Mild (scores 10–13)–labelled as 1
- Moderate (scores 14–20)–labelled as 2
- Severe (scores 21–27)–labelled as 3
- Extremely Severe (scores 28+)–labelled as 4

### 2.2 Experimental design

#### 2.2.1 Survey objective

The primary objective of this survey was to assess the mental condition of the students due to their increased exposure to digital tools, mobile connectivity and internet activities associated with the hybrid or distance mode of learning. Besides, sleep data and internet connectivity status were also collected to see whether there was an irregular connection between the hybrid mode of learning and physical activity.

As a general guideline, participants were instructed to not think about a question for too long and all the items related to the mental condition were to be filled in based on the experience of the past 4 weeks. At the end of the survey, a total of 444 responses were submitted and the data was then saved into a.csv file for further processing.

#### 2.2.2 Exploratory data analysis

Of the 444 respondents, 291 were male and 153 were female participants ranging from below 20 years to over 35 years of age. University students usually enroll after the age of 17 years. Thus ‘below 20 years’ implies the range between 17 to 20 years. The survey was primarily focused on the students at the Islamic University of Technology (IUT) situated in Gazipur, Bangladesh. As a result, most of the participants were aged between 21 to 25 years. Besides, since the university operates under the umbrella of an international organization named Organization of Islamic Cooperation (OIC); international students from more than 20 countries such as Iran, Pakistan, Brunei, Indonesia, Uganda, Nigeria, Cameroon, Gambia, Yemen and so on were attending this university under this international banner. These participants particularly have experienced their academic activities being transitioned from remote learning to hybrid mode. Moreover, the participants ranged from undergraduate-first year to postgraduate studies and from several types of contexts (e.g., small towns, big cities), which added to the authenticity of the results obtained through the survey. First-year students were comparatively higher in number than the other four academic levels and the significantly higher number of respondents came from big cities rather than from villages or small towns. The overall data distribution is shown in **Tables 1**–**5**.

**Table 1:** The male-to-female ratio of the study group.

Participant	Number	Percentage of total (est.)
Male	291	65.5%
Female	153	34.5%

**Table 2:** The age range of the study group.

Participant	Number	Percentage of total (est.)
Below 20 years	132	30.18%
Between 21 to 25 years	271	61.03%
Between 26 to 30 years	27	6.08%
Between 31 to 35 years	8	1.80%
Above 35 years	6	1.35%

**Table 3:** Ethnicity/geographical location of the study group.

Participant	Number	Percentage of total (est.)
South Asia (e.g., Bangladesh, Pakistan, Afghanistan, and Maldives)	365	82.21%
Southeast Asia (e.g., Malaysia, Indonesia, and Brunei)	30	6.76%
Africa (e.g., Uganda, Nigeria, Gambia, Cameroon, and Egypt)	44	9.91%
Others (e.g., Turkey, Saudi Arabiya, Iran, Yemen, and UAE)	5	1.12%

**Table 4:** Academic level of the study group.

Participant	Number	Percentage of total (est.)
Undergraduate–First year	179	40.31%
Undergraduate–Second year	57	12.83%
Undergraduate–Third year	92	20.72%
Undergraduate–Fourth year	66	14.86%
Postgraduate	50	11.26%

**Table 5:** Residence of the study group.

Participant	Number	Percentage of total (est.)
Village / Rural Area (<300 inhabitants per km^2^)	14	3.15%
Small City / Town (300 to 1500 inhabitants per km^2^)	98	22.07%
Big City (>1,500 inhabitants per km^2^)	332	74.77%

An elaborated analysis with the findings of the relationship between survey data and depression severity is demonstrated in the next section as stated in the research contribution.

### 2.3 Data pre-processing

Before training different algorithms with the dataset, several pre-processing steps need to be performed. For calculating the mean and standard deviation values from DASS-Depression scale, Microsoft Excel was used to perform descriptive statistics. All the coding simulation was executed on the Jupyter Notebook of the Python Navigator platform. At first, the input-output was separated, and some irrelevant columns were dropped. The dropped columns’ presence was not affecting the accuracy value in one way or the other, for which they were eliminated in favor of improving accuracy, F1-score, and ROC-AUC. In the.csv file, the ‘Result’ column was used as output. There were 41 columns in the preprocessed dataset. 3 columns were dropped from the dataset which are the timestamp, voluntary consent, and sum. The remaining 38 columns were used as input. Then, alphabetical data were converted into numerical values using label encoding. It refers to converting the labels into a numeric form to convert them into a machine-readable form. In this way, ML classifiers can decide in a better way about how those labels must be encoded without increasing the dataset dimensions [70]. It is an important pre-processing step for the structured dataset in supervised learning. After that, the MinMaxScaler from scikit-learn library was applied to the dataset directly to normalize the input variables.

MinMaxScaler scales all the data features in the range [0, 1] if all data are non-negative, otherwise in the range [–1, 1] if there are negative values in the dataset. This scaling compresses all the inliers in the narrow range [0, 1]. As there were no negative values in the dataset, the default configuration was utilized and scaled values were obtained in the range [0, 1]. When the upper and lower boundaries are well defined from domain knowledge, MinMaxScaler is typically utilized [71]. One striking feature of the MinMaxScaler is that it preserves the shape of the original distribution without tampering with the information included in the original data. It should be noted that the MinMaxScaler does not lessen the significance of outliers. The feature returned by the MinMaxScaler has a default range of values between 0 and 1. Whenever the data possess a bounded range of values or the distribution is not Gaussian, the MinMaxScaler is widely adopted [72]. As the dataset of this study was also not following the Gaussian distribution, the Min-Max method was adopted for feature scaling.

First, a MinMaxScaler instance was defined with default hyperparameters. Once defined, the *fit_transform()* function was utilized to generate a transformed version of the dataset.

### 2.4 Feature engineering

After the pre-processing of the dataset, the methodology was divided into two parts. In the first part, all 38 features had been used to train the eight ML models named previously without including a feature selection or feature extraction step. However, to determine how substantial this automated approach was in terms of performance, several feature engineering methods were added in the second part to see the performance change. The adopted feature selection methods were the Chi-square test and RFE, whereas the selected feature extraction methods were PCA and SparsePCA [73, 74]. These methods were yet to be tested in the literature unlike some other methods such as SelectKBest, and Boruta [61, 63] and hence, this study was aimed to investigate the difference in the results among them.

#### 2.4.1 Feature selection methods

Feature selection methods select the most apposite features by disregarding the irrelevant ones so that the ML models do not suffer from mediocre performance on high-dimensional data [75]. It is also more interpretable to the researchers as it reduces the training time. In this study, 30 features out of 61 features were selected using both the Chi-square test and RFE method which are discussed below.

*Chi-square test*. In the Chi-square (*χ*^2^) test, the observations are categorized into classes that are mutually exclusive in conventional applications. The test statistic generated from the observations follows a *χ*^2^ frequency distribution if the null hypothesis, that there are no significant differences between the classes in the population, is true. A random sample of n observations from a population is divided into k mutually exclusive classes, each with a set of observed numbers, *x*_*i*_ (for *i* = 1,2,…,*k*). The dependency of two events is tested by using this formula [75]:

$$\chi^{2}=\sum_{i=1}^{k}\frac{{\left(x_{i}-m_{i}\right)}^{2}}{m_{i}}$$ (1)

where, *x*_*i*_ = observed value, and expected value, *m*_*i*_ = *np*_*i*_, *n* being the number of samples. And,

$$\sum_{i=1}^{k}p_{i}=1$$ (2)

Here, high *χ*^2^ value means the feature set has significant relevance with the output and thus that feature set can be used for the training of the ML models.

*Recursive Feature Elimination (RFE)*. RFE method falls under the filtering category of using a proxy measure to evaluate a feature subset. In place of a clear best feature subset, many filters offer a feature ranking, with cross-validation used to determine the ranking’s cutoff point. RFE is one such method which keeps only the best key features by calculating the weights of each feature for a given output. As a result, the model complexity gets much reduced and can be run efficiently by training on an optimized set of features. The weighted voting scheme follows this equation [76]:

D(x)=w.(x−μ) (3)

where, *D*(*x*) = decision function, *w* = weight vector and *μ* = is the mean vector over all training patterns. The ranking is determined by using the following equation in an iterative process:

$$DJ(i)=\frac{1}{2}\frac{d^{2}J}{dw_{i}^{2}}{\left(Dw_{i}\right)}^{2}$$ (4)

where, *J* = cost function. The iterative algorithm is as follows:

1. Train the classifier first by optimizing the weights *w*_*i*_ with respect to *J*.
2. Determine the *DJ*(*i*) or (*w*_*i*_)^2^ ranking criterion for each feature.
3. Discard the component with the lowest ranking criterion.

#### 2.4.2 Feature extraction methods

In the case of feature extraction, a new set of features is used for model training, which are extracted from the existing features. The outcome is similar to feature selection, that is, a reduced and concise set of features capable of predicting the outcome most precisely. The methods also help to save memory and computational power while producing adequate performance. Though feature extraction methods are vastly employed for image processing purposes, discrete valued features can be tested as well to explore their effectiveness [77]. The feature extraction methods applied in this study were PCA and SparsePCA, both of which are discussed below:

*Principal Component Analysis (PCA)*. PCA is a classical statistical method for transforming attributes of a dataset into a new set of uncorrelated attributes called principal components. PCA can be used to reduce the dimensionality of a dataset, while still retaining the variability of the dataset as much as possible. In PCA, a matrix *X*, which is a *N*×*p* matrix, serves as the dataset’s representation. All the observations for one attribute are contained in each column, *X*_*j*_. The set of inputs *X*_1_,*X*_2_,…,*X*_*n*_ is transformed by PCA into another set of column vectors *T*_1_, *T*_2_,…,*T*_*N*_. A *p*×*p* matrix *P* specifies this linear transformation of the matrix *X* such that the transformed variables *T* are given by:

T=XP (5)

Alternatively, the equation can be rewritten as:

$$X=TP^{T}$$ (6)

where *P* is known as the loading matrix, and columns of this matrix can be calculated as eigenvectors of the matrix *X*^*T*^*X* [77].

*Sparse Principal Component Analysis (SparsePCA)*. By adding sparsity structures to the input variables, SparsePCA expands the traditional PCA approach for reducing the dimensionality of data. The principal components of standard PCA are often linear combinations of all input variables, which is a particular drawback. By identifying linear combinations with a smaller number of input variables, SparsePCA gets around this drawback [78]. The sparse factors that account for the greatest amount of variance can be expressed as follows:

$$\varphi (\rho )=z^{T}\sum z-\rho Card(z)$$ (7)

where, *z*∈*R*^*n*^, *ρ* = parameter controlling sparsity, *Card*(*z*) = the number of non-zero coefficients of *z*. There are several solutions to this problem, one of which is Greedy Search Algorithm [78]. For this algorithm, the input would be ∑∈*R*^*n*×*n*^. The steps are as follows:

1. Preprocessing: Sort variables using diagonal elements in decreasing order, then permute ∑ elements, as required. Perform the Cholesky decomposition calculation ∑ = *A*^*T*^*A*.
2. Initialization: $I_{1}=\{1\},\;x_{1}=\frac{a1}{\left|\left|a1\right|\right|}$
3. Compute $i_{k}=argmax_{i\notin I_{k}}\lambda_{max}(\sum_{j\in I_{k}\cup \{i\}}a_{j}a_{j}^{T})$
4. Set $I_{k+1}=I_{k}\cup \{i_{k}\}$ and compute *x*_*k*+1_ as the leading eigenvector of $\sum_{j\in I_{k+1}}a_{j}a_{j}^{T}$.
5. Set *k* = *k*+1. If *k*<*n*, go back to step 3.

### 2.5 Classification models

Before building the ML models, 5-fold cross-validation was performed on the train and test set. In this way, the dataset was divided into 5 distinct segments in the first of which 80% of the dataset was used for training and the remaining 20% was used for testing. For the next segment, the 20% testing set used in the first segment got included in the 80% training set and another 20% set, which was not part of the testing dataset in the first segment, was used for the evaluation step. This process was repeated 5 times in total independently to ensure that one specific set does not get selected for training and simultaneously, only a specific set is not used for testing purposes. As a result, the possibilities of overfitting issues were minimized and the mean value of the 5 folds was taken as the final score for each of the 3 metrics.

Thus, the previously mentioned 8 ML models were trained using the training set and the models’ performance was evaluated using the test set. A brief description of each of the models is given below:

#### 2.5.1 Gaussian Naïve Bayes

It is a classifier that uses only probabilistic models and the Bayes theorem. The fundamental presumption in this situation is that the features for which an outcome is anticipated are independent of one another [79]. The following is the classifier formula:

$$y=argmax_{y}p(y)\prod_{i=1}^{n}P(x_{i})|y$$ (8)

The probability estimate will be zero if a feature never appears in any categories in the training data [54]. This happens because the probability estimate is related to the number of times a feature’s value occurs.

#### 2.5.2 Logistic Regression

The "logistic function" is used as the cost function in this algorithm, which is based on the idea of probability. The algorithm’s nature limits this cost function to a range between 0 and 1, and its formula is as follows:

$$\theta (x)=\frac{1}{1+e^{-(\beta_{0}+\beta_{1}X)}}$$ (9)

To distinguish one class from another in a classification task, a threshold probability value is specified [80]. The same general idea applies to multiclass regression, with the exception that there are M possible outcomes rather than simply two.

#### 2.5.3 Random Forest

The decision tree concept is used in random forests, which is extended further by incorporating many different individual trees. A weight is assigned to each of these trees’ results, and the sum of those weights serves as the foundation for determining the anticipated class through voting. The bagging formula is as follows:

$$f=\frac{1}{B}\sum_{b=1}^{B}f_{b}(x\prime )$$ (10)

where, *f*_*b*_ = classification tree, *B* = number of bagging (1, 2…, b), *x*′ = training set. This algorithm offers more accuracy compared to other classification techniques because it is an ensemble classifier that takes help of multiple decision trees [81].

#### 2.5.4 Support Vector Machine (SVM)

This technique locates a hyper-plane that divides a collection of points in an N-dimensional space depending on features. To secure the classification process for the future, the hyper-plane must maximize the margin between the classes of data. Support vectors are the values closest to the hyper-plane in this case, impacting the margin’s position and orientation. The equation for linear SVM, among other types, is as follows:

$$w^{T}*x-b=0$$ (11)

Here, *w*^*T*^ = normal vector to the hyperplane, and *b* determines the offset distance from the origin. SVM has the advantage of being less prone to the over-fitting issue than other algorithms [82].

#### 2.5.5 Light Gradient Boosted Machine

Shortened as LightGBM, this algorithm is developed using a decision tree algorithm and has the ability to reproduce results like a boosting algorithm (XGBoost). Regarding efficiency and memory usage, LightGBM employs a highly efficient decision tree learning technique based on histograms. As an ensemble algorithm, any arbitrary differentiable loss function and the gradient descent optimization procedure are used to fit the models. A few steps of the algorithm Gradient-based One-Side Sampling technique used in LightGBM is given below [83]:

1. Input: training data (I), iterations (d), sampling ratio of large gradient data (a) and small gradient data (b), loss function (L)
2.
$models\leftarrow \{\},fact\leftarrow \frac{1-a}{b}$

3.
topN←a×len(I),randN←b×len(I)

4. For *d* iterations, newModel←L(I[topSet+randSet],−g[topSet+randSet]); where, topset=sorted[1:topN], randSet=RandomPick(sorted[topN:len(I)],randN)

#### 2.5.6 Multi-layer Perceptron

Multi-layer Perceptron or MLP performs similarly to a supervised learning method by adding backpropagation during training. Specifically, this feedforward network requires at least three layers of nodes namely, the input layer, hidden layer, and output layer. One of the activation functions utilizing the hyperbolic tangent function is as follows:

$$y(v_{i})=tanh\;(v_{i})$$ (12)

Here, node output is represented by *y* and *v* = weighted sum of the inputs. The node weights that are to be modified in the backpropagation process in accordance with the change that reduces overall output error, follows this equation [84]:

$$\varepsilon (n)=\frac{1}{2}\sum e_{j}^{2}(n)$$ (13)

where, *e*_*j*_ = degree of error in an output node *j* while *n* represents a datapoint.

#### 2.5.7 CatBoost

Another boosting framework that has been used in this study is CatBoost which is one of the most frequently used ML models in the modality. In contrast to LightGBM which has a probability of losing information for clustering tail categories according to expected target value (target statistics), CatBoost uses the target statistics as new numerical features. These features follow an ordering principle in which a random permutation is introduced for the training samples and the preceded memory is used to calculate target statistics [85]. An example of a training and a testing sample are as follows:

$$D_{k}=\{x_{j}:\sigma (j)<\sigma (k)\};D_{k}=D$$ (14)

where, *σ* signifies a random permutation of *j* or *k* samples to satisfy the target statistics equation.

#### 2.5.8 K-Nearest Neighbor (KNN)

Finding the data points that are close to one another is how the KNN algorithm operates. To compare a new point to k numbered reference points in order to establish how close it is, Euclidean distance is typically utilized. The k value in this case determines the amount of stability the algorithm will maintain in precise predictions [86]. A point in a feature space that should be given to the category of closest neighbors is:

$$C_{n}(x)=Y_{1}$$ (15)

where, *x* = a point in space, and *Y*_1_ = a class the point is assigned to. It is more straightforward to construct compared to other models because no additional suppositions or adjusting are required.

The parameters used for each of the classifiers can be seen in Table 6. Other than the LightGBM and Catboost classifiers, which have their own libraries, scikit-learn was the library to call the parameters and to set the values accordingly. All the features of engineering methods followed these values.

**Table 6:** Parameter names and associated values for the ML models.

No.	Classifier Name	Parameters	Value
1.	Gaussian Naïve Bayes	var_smoothing	1e-09, 1e-08, 1e-07
2.	Logistic Regression	solver	liblinear, newton-cg
		random_state	0,1
		C	0.1, 1, 10
		tol	0.01, 0.001, 0.0001, 0.1
3.	Random Forest	n_estimators	50, 100, 75, 200, 300, 500
		min_samples_split	2, 4, 8, 10
4.	Support Vector Machine	random_state	0, 1
		C	1, 5, 7, 10, 12, 20, 28
		degree	1, 2, 3, 4
5.	Light Gradient Boosted Machine	reg_alpha	0.0, 0.1, 1, 5, 3
		n_estimators	1, 2, 10, 50, 100
		reg_lambda	0.0, 0.1
		learning_rate	0.1, 0.5, 0.01
6.	Multi-layer Perceptron	hidden_layer_sizes	10, 100, 300, 500, 800
		alpha	0.0001, 0.3, 0.001, 0.1, 0.5, 1, 3, 5
		random_state	0, 1
		tol	0.0001, 0.01, 0.001, 0.1, 1, 10, 50
7.	CatBoost	eta	0.3, 0.001, 0.1, 0.5, 1, 3, 5
		n_estimators	1, 2, 10, 50, 100, 75, 200
		reg_lambda	0.1, 1, 5, 3, 0.5
8.	K-Nearest Neighbor	n_neighbors	1, 2, 3, 4, 5
		leaf_size	15, 30, 50, 100
		p	1, 2, 3

### 2.6 Hyperparameter optimization

**Fig 1:**
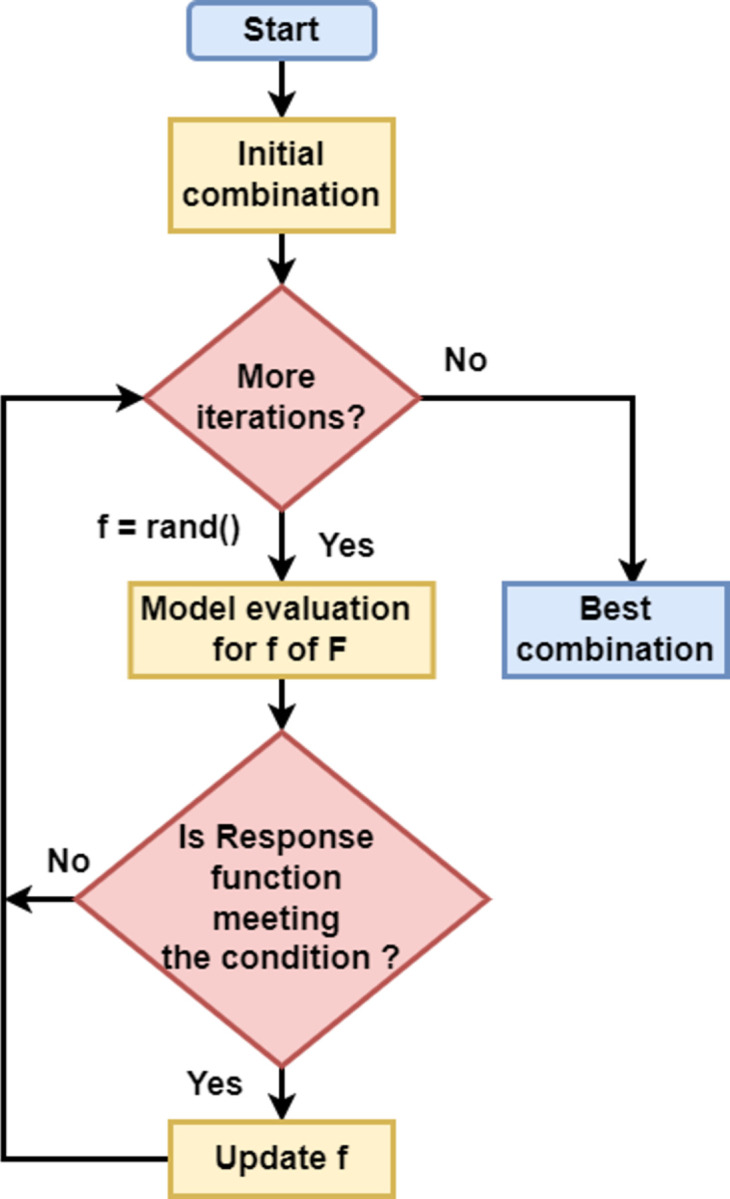
Random search hyperparameter optimization.

For achieving better results, RandomSearchCV was employed for optimizing the hyperparameters for all eight classifiers. In Random Search, a random combination of hyperparameter values is used to train the model, which performs excellently for discovery and obtaining the combinations that are hard to predict [87]. Unlike Grid-search which frequently takes more time to complete as it tests every combination, Random Search tends to be efficient most of the time. The hyperparameter optimization problem can be addressed in the following way:

$$f^{\left(*\right)}=argmin_{f\in F}\psi (f)$$ (16)

where, *f* = hyperparameter, *F* = set of trial values (*f*^1^,…,*f*^*S*^), *ψ* = hyperparameter response function. A flowchart for finding the optimal combination of hyperparameter in random search is given in Fig 1:

This step is an experimentation step for the study participants in this study and so, the hyperparameter combination can be different for other study participants.

### 2.7 Performance analysis

The metrics that were considered to evaluate the ML classifiers were accuracy, F1-score, and ROC-AUC. These metrics illustrate the performance of the ML models holistically. The confusion matrix is also another way to show the results in terms of distinct numbers.

#### 2.7.1 Accuracy

This metric is the most widely used metric to measure the correctness of the ML model, that is, the proportion of accurately anticipated observations to all observations. Although accuracy is one of the mostly used performance metrics in the field of ML, two more metrics namely, F1 and ROC-AUC scores are also adopted by the researchers to evaluate the outcome of an algorithm [88]. The formula for accuracy is:

$$Accuracy=\frac{TP+TN}{TP+FP+TN+FN}$$ (17)

Here, TP and TN denote the number of positive and negative instances that are accurately classified in the multi-class classification of this study. In this type of classification, a threshold is set for each class and any prediction that is out of the threshold falls into a misclassification.

#### 2.7.2 F1-score

This metric displays the precision and recall weighted average and the F1-score is measured to balance recall and precision scores [89]. Moreover, the value range of the F1-score is between 0 to 1.0 which can be calculated using Eq (18).


$$F1=\frac{2*Recall*Precision}{Recall+Precision}$$ (18)

Here,

$$Recall=\frac{TP}{TP+FN}$$

$$Precision=\frac{TP}{TP+FP}$$

#### 2.7.3 ROC-AUC

ROC-AUC is the value of the area under a Receiver Operating Characteristic (ROC) curve which is calculated using True Positive Rate (TPR) and False Positive Rate (TPR) obtained by Eqs (19–20)

$$TPR=\frac{TP}{TP+FN}$$ (19)

$$FPR=\frac{FP}{FP+TN}$$ (20)

As opposed to a threshold-based metric like accuracy, the AUC value indicates a classifier’s performance by using ranking, and sometimes, it exhibits a better metric for evaluation [90]. For the multi-class classification task, the performance is measured using this formula [91]:

$$M=\frac{2}{c(c-1)}\sum_{i<j}\hat{A}(i,j)$$ (21)

where, $\hat{A}$ is the probability operator, *i* and *j* is a pair of classifiers, *c* is the number of classes. In general, the one vs. all approach can be utilized to plot N number of AUC curves for N number of classes in a multi-class classification problem [92].

**Fig 2:**
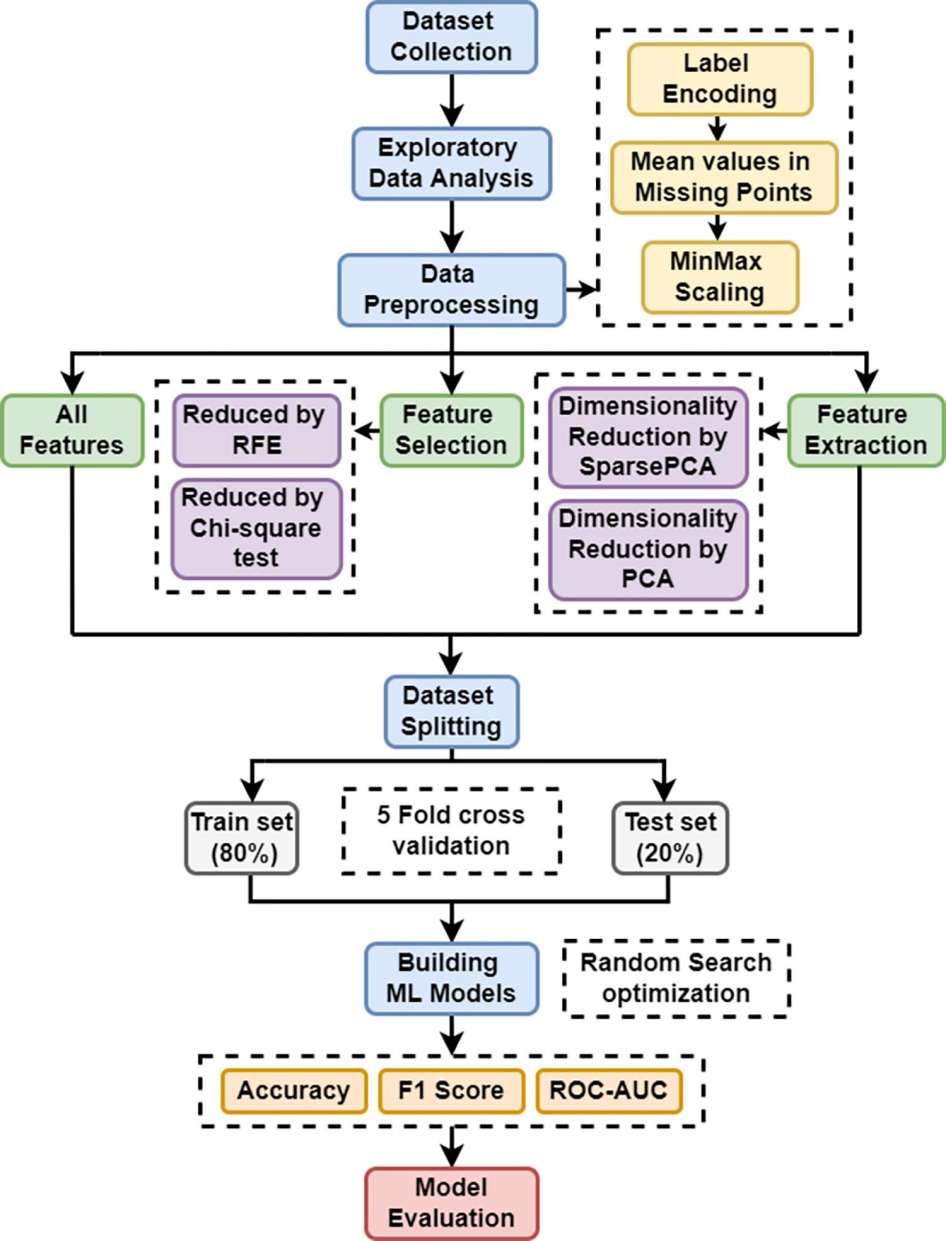
Overall workflow diagram.

Lastly, an overall workflow diagram is shown in Fig 2, and all the findings are exhibited and conscientiously analyzed in the following section.

## 3 Results

### 3.1 Digital well-being analysis

#### 3.1.1 Gender, age-groups, and ethnicity-based depression

An in-depth analysis was performed to better understand the connection between depression severity and the different study groups. Here, all the illustrations have been shown using boxplots from the Python matplotlib library and seaborn package. A boxplot is a graphical method of showing the data distribution in terms of range, locality, and skewness by dividing the amount of data into four quartiles [93]. In the graph, the box depicts the data from the first quartile to the third quartile with a whisker (the line protruding from the sides of each box) pointing out the minimum and maximum values, if applicable.

**Fig 3:**
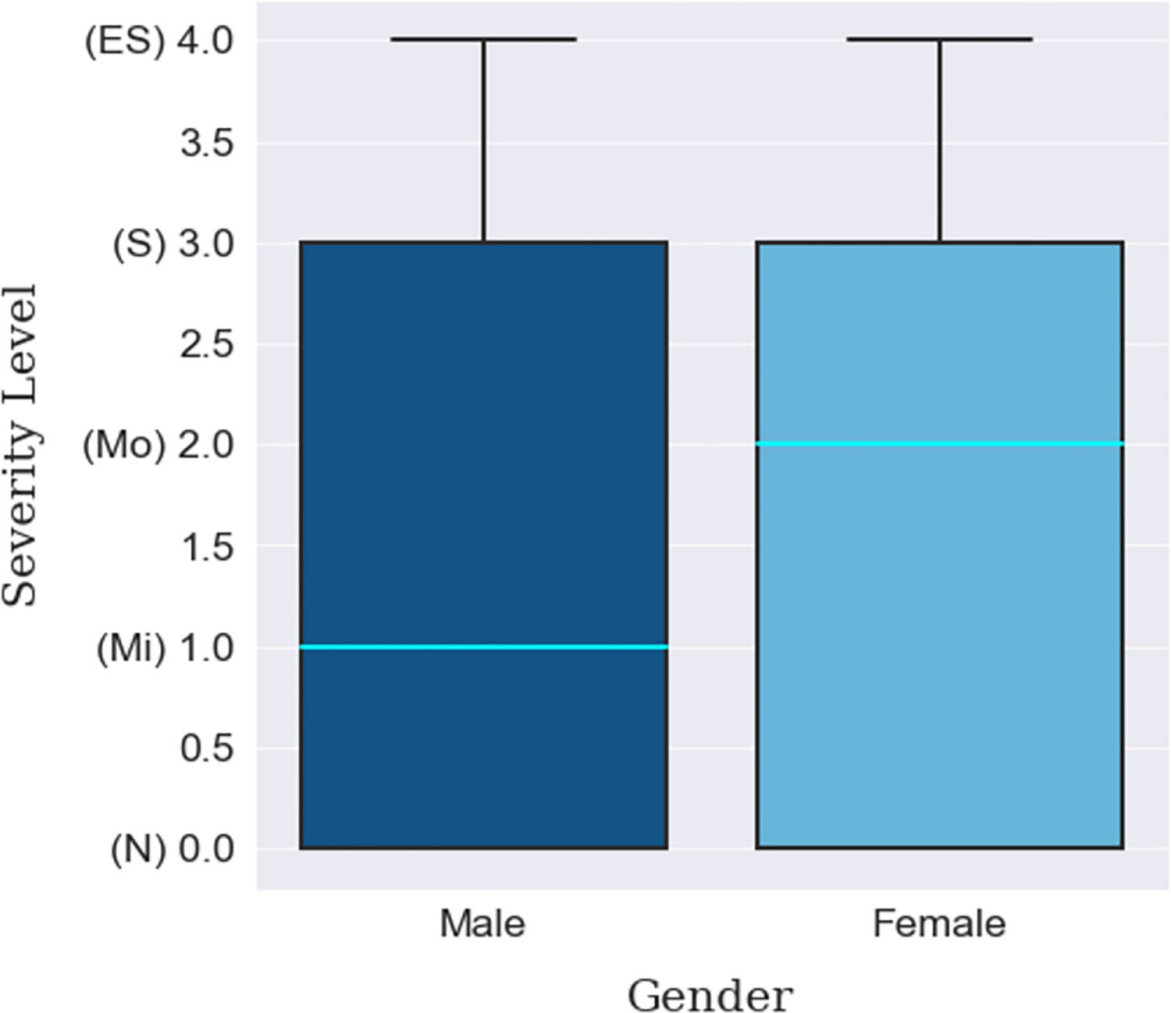
Gender-based depression severity distribution.

**Fig 4:**
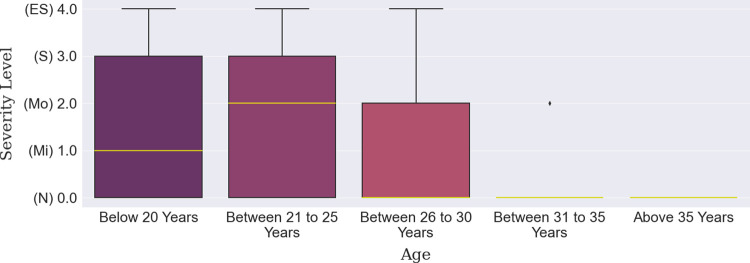
Age-based depression severity distribution.

**Fig 5:**
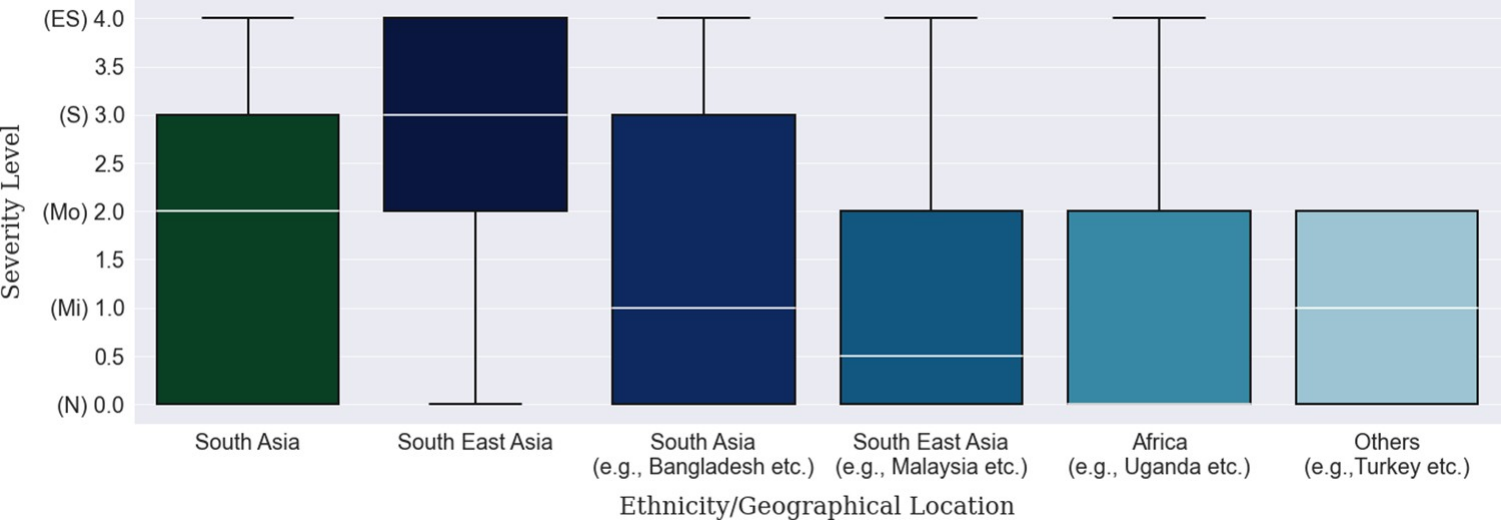
Ethnicity-based depression severity distribution.

Firstly, there were about twice as many data points for male participants as for female participants, as shown in Table 7. When it comes to depression levels, more than half of the data points from females gathered under the ‘Moderate’ and ‘Extremely Severe’ categories compared to that of the males, as shown by the median line in Fig 3. Overall, no depression was observed among about 44% of the accumulated participants while mild-to-moderate and severe-to-extremely-severe depression were observed among 25% and 31% of the population, respectively. For the age groups, it can be seen from **Table 8** that there was a rise in the ‘Extremely Severe’ level for the participants between 21 to 25 years. The median line shown in Fig 4 also demonstrates the relatively high level of depression in this group compared to two other groups which are below 20 years and participants between 26-to-30-year. Lastly, though a large number of participants were inhabitants of Bangladesh, there were international participants whose depression severity can be observed in Tables 9 and 10. South Asian participants from countries other than Bangladesh, Pakistan, and Afghanistan. were in a state of moderate depression compared to mild depression of these countries’ participants. Notable mention here is that most participants from the Southeast Asian countries fell under the ‘Moderate’ to ‘Extremely Severe’ category and as a result, the boxplot (Fig 5) has been shifted up to those levels., The least amount of depression can be seen in the participants from Africa, more than two-thirds of whom were facing no depression.

**Table 7:** Result of the data analysis among males and females.

Depression Severity Level	Male		Female		Percentage of total
	Count, N = 291	DASS-Depression Score (Mean ± Deviation)	Count, N = 153	DASS-Depression Score (Mean ± Deviation)	
Normal (N)	133	2.27 ± 2.75	62	2.74 ± 2.96	44%
Mild (Mi)	29	10.89 ± 1.01	11	11.09 ± 1.04	25%
Moderate (Mo)	41	16.48 ± 2.35	30	15.73 ± 1.79	
Severe (S)	36	23.83 ± 1.61	16	23.75 ± 1.61	31%
Extremely Severe (ES)	52	34 ± 5.13	34	35.17 ± 5.68	

**Table 8:** Result of the data analysis among age groups.

Depression Severity Level	Below 20 years		Between 21 to 25 years		Between 26 to 30 years		Between 31 to 35 years		Above 35 years	
	Count, N = 132	DASS-Depression Score (Mean ± Deviation)	Count, N = 271	DASS-Depression Score (Mean ± Deviation)	Count, N = 27	DASS-Depression Score (Mean ± Deviation)	Count, N = 8	DASS-Depression Score (Mean ± Deviation)	Count, N = 6	DASS-Depression Score (Mean ± Deviation)
Normal (N)	52	2.19 ± 2.77	112	2.76 ± 2.95	18	1.77 ± 2.55	7	1.42 ± 1.51	6	1 ± 1.67
Mild (Mi)	24	10.83 ± 1.01	15	11.06 ± 1.03	1	12 ± 0	0	-	0	-
Moderate (Mo)	16	15.62 ± 1.96	51	16.43 ± 2.23	3	14.66 ± 1.54	1	16 ± 0	0	-
Severe (S)	19	24.10 ± 1.69	31	23.74 ± 1.52	2	22 ± 0	0	-	0	-
Extremely Severe (ES)	21	35.04 ± 5.74	62	34.16 ± 5.28	3	36.67 ± 4.61	0	-	0	-

**Table 9:** Result of the data analysis among ethnicity groups (South Asia and Southeast Asia).

Depression Severity Level	South Asia		Southeast Asia		South Asia (e.g., Bangladesh, Pakistan, Afghanistan, and Maldives)	
	Count, N = 41	DASS-Depression Score (Mean ± Deviation)	Count, N = 22	DASS-Depression Score (Mean ± Deviation)	Count, N = 324	DASS-Depression Score (Mean ± Deviation)
Normal (N)	16	2.5 ± 2.78	5	3.6 ± 4.09	136	2.46 ± 2.79
Mild (Mi)	2	12 ± 0	0	-	36	10.94 ± 1.01
Moderate (Mo)	8	16.75 ± 2.12	5	15.2 ± 1.78	50	16.24 ± 2.12
Severe (S)	5	23.6 ± 1.67	2	23 ± 1.41	41	23.85 ± 1.63
Extremely Severe (ES)	10	34.8 ± 5.01	10	35 ± 4.92	61	34.32 ± 5.63

**Table 10:** Result of the data analysis among ethnicity groups (Southeast Asia, Africa and Others).

Depression Severity Level	Southeast Asia (e.g., Malaysia, Indonesia, and Brunei)		Africa (e.g., Uganda, Nigeria, Gambia, Cameroon, and Egypt)		Others (e.g., Turkey, Saudi Arabiya, Iran, Yemen, and UAE)	
	Count, N = 8	DASS-Depression Score (Mean ± Deviation)	Count, N = 44	DASS-Depression Score (Mean ± Deviation)	Count, N = 5	DASS-Depression Score (Mean ± Deviation)
Normal (N)	4	5 ± 3.46	32	1.5 ± 2.43	2	7 ± 1.41
Mild (Mi)	1	10 ± 0	0	-	1	10 ± 1
Moderate (Mo)	2	20 ± 0	4	14.5 ± 1	2	14 ± 0
Severe (S)	0	-	4	24 ± 1.63	0	-
Extremely Severe (ES)	1	30 ± 0	4	35.5 ± 4.43	0	-

#### 3.1.2 Academic level-based depression

**Fig 6:**
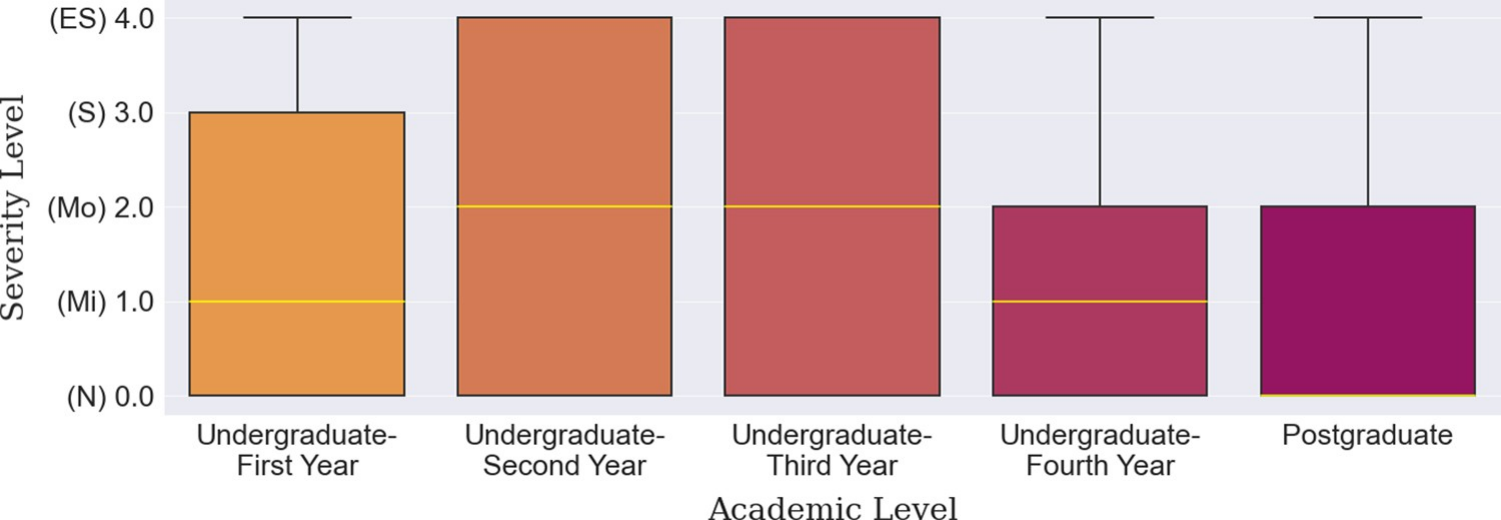
Academic level-based depression severity distribution.

**Fig 7:**
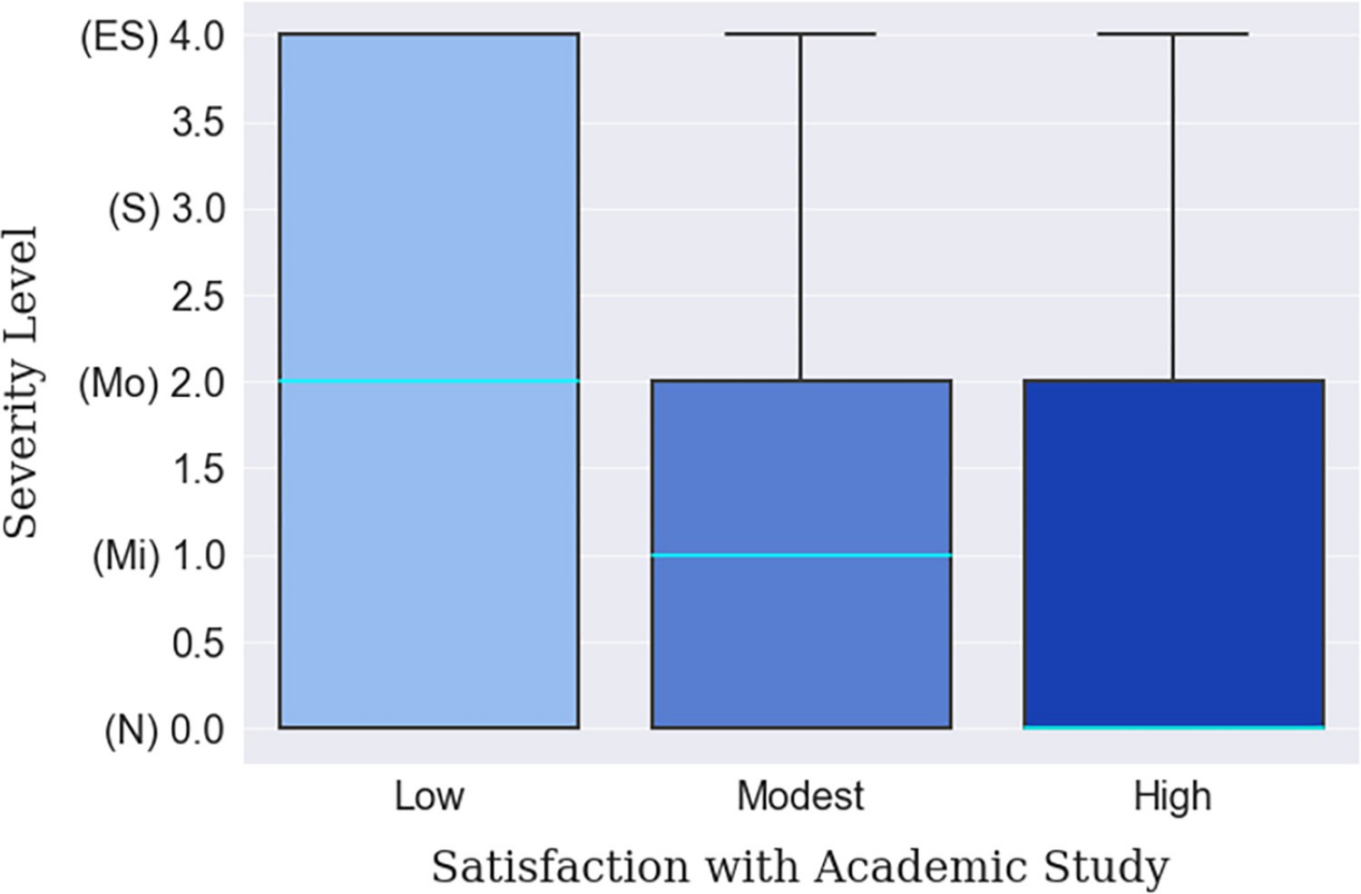
Satisfaction with academic study-based depression severity distribution.

The prevalence of depression among the students of different academic levels ranging from first-year students to postgraduate was identified; the results are presented in Figs 6, 7 and Table 11. It was found that a randomly selected student in his/her 2^nd^ or 3^rd^ academic year has a larger probability of facing a higher severity of depression than a 1^st^ year or postgraduate student, as shown in Fig 6. Postgraduate students particularly experienced the least amount of depression during the hybrid mode of learning (median line at ‘Normal’), immediately followed by undergraduate-fourth year students (median line at ‘Mild’). This can be counted as a valid comparison as the percentages of the four academic level students excluding the first year were relatively adjacent to each other (Table 11). Meanwhile, one finding also suggests that if the students were satisfied with their examination results and other academic activities, they fell under the ‘Normal’ category more than the students who were dissatisfied. In fact, half of the students who were not enjoying the academic curriculum were at the ‘Moderate’ depression level contrary to half of the contented students who experienced no depression at all (Fig 7). This is a crucial finding since this study was intended to investigate the applicability of hybrid learning which should be able to cater for a broad student demographic.

**Table 11:** Result of the data analysis among academic level groups.

Depression Severity Level	Undergraduate–First year		Undergraduate–Second year		Undergraduate–Third year		Undergraduate–Fourth year		Postgraduate	
	Count, N = 179	DASS-Depression Score (Mean ± Deviation)	Count, N = 57	DASS-Depression Score (Mean ± Deviation)	Count, N = 92	DASS-Depression Score (Mean ± Deviation)	Count, N = 66	DASS-Depression Score (Mean ± Deviation)	Count, N = 50	DASS-Depression Score (Mean ± Deviation)
Normal (N)	74	2.59 ± 2.78	20	3.7± 3.13	36	2.05 ± 2.63	32	2.31 ± 3.13	33	1.75 ± 2.43
Mild (Mi)	25	10.88 ± 1.01	3	10.67 ± 1.15	4	11 ± 1.15	7	11.14 ± 1.06	1	12 ± 0
Moderate (Mo)	23	16.17 ± 2.08	10	16.8 ± 2.69	17	15.88 ± 2.05	11	16.36 ± 2.33	10	15.8 ± 1.98
Severe (S)	27	23.77 ± 1.69	7	24 ± 1.63	11	23.81 ± 1.66	4	24.5 ± 1	3	22.67 ± 1.15
Extremely Severe (ES)	30	34.4 ± 5.71	17	33.88 ± 5.21	24	35.16 ± 5.07	12	33.5 ± 5.85	3	36.67 ± 4.61

#### 3.1.3 Residence and physical activity-based depression

**Fig 8:**
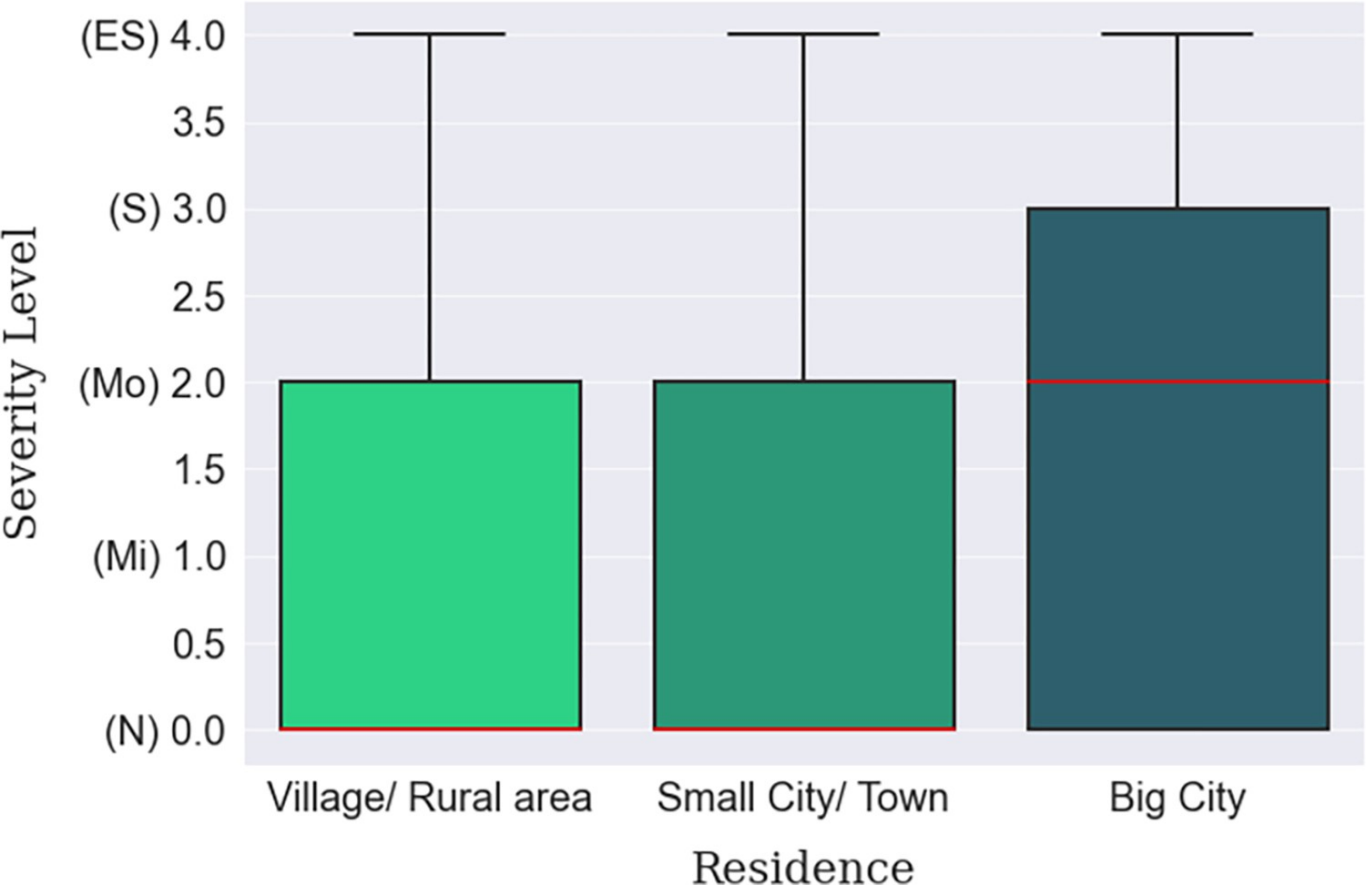
Residence-based depression severity distribution.

**Fig 9:**
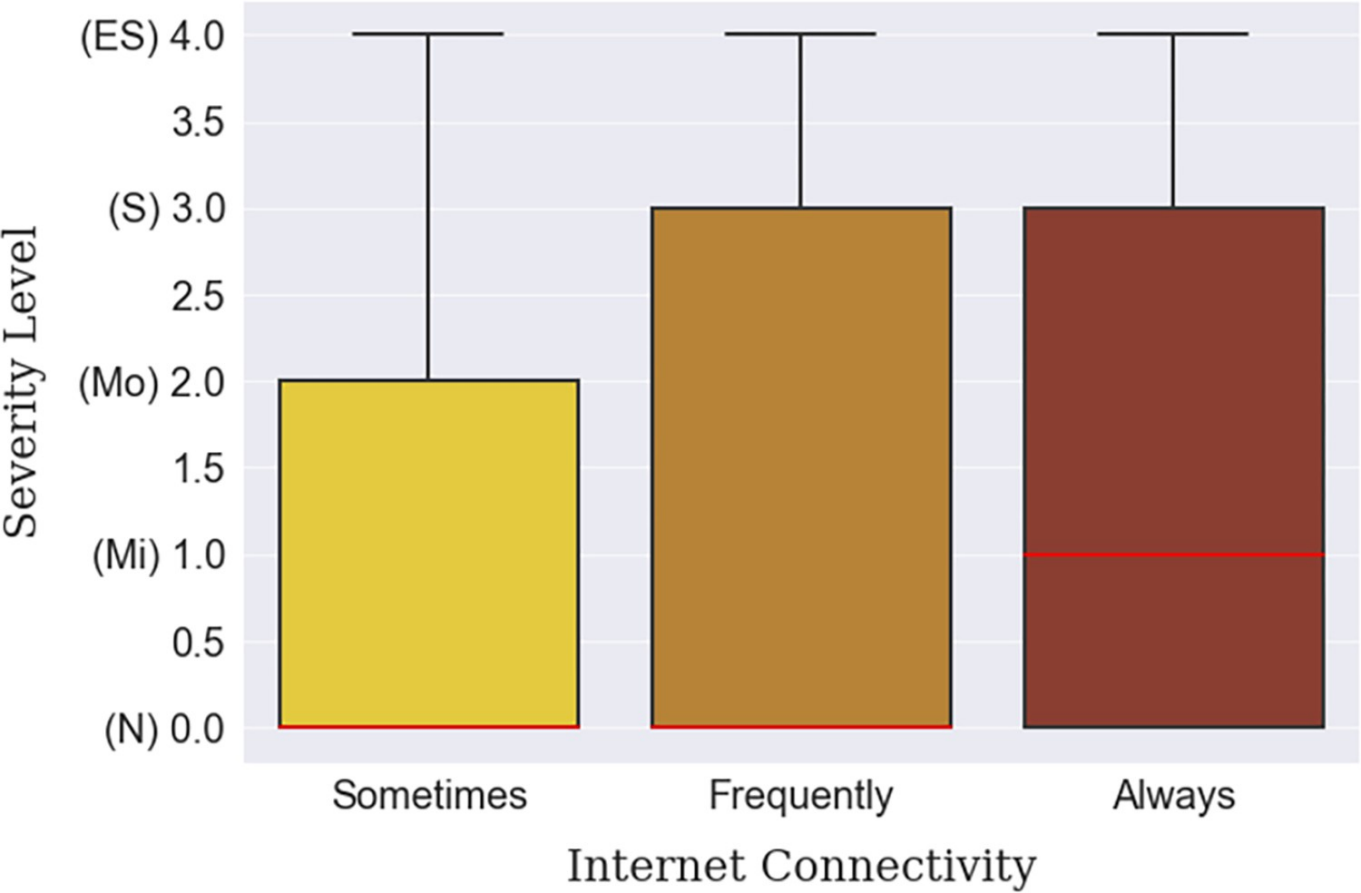
Internet connectivity-based depression severity distribution.

**Fig 10:**
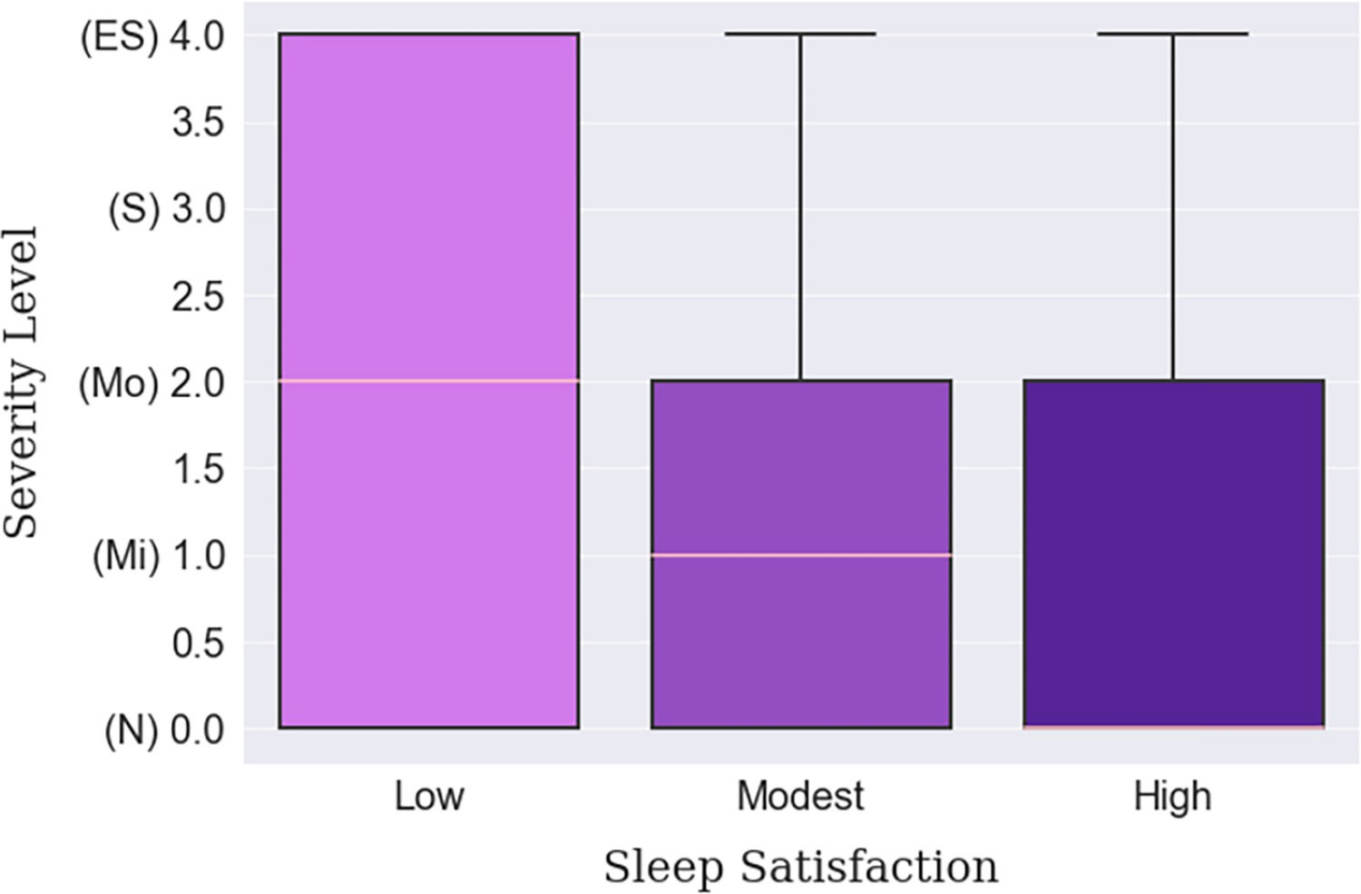
Sleep satisfaction-based depression severity distribution.

**Fig 11:**
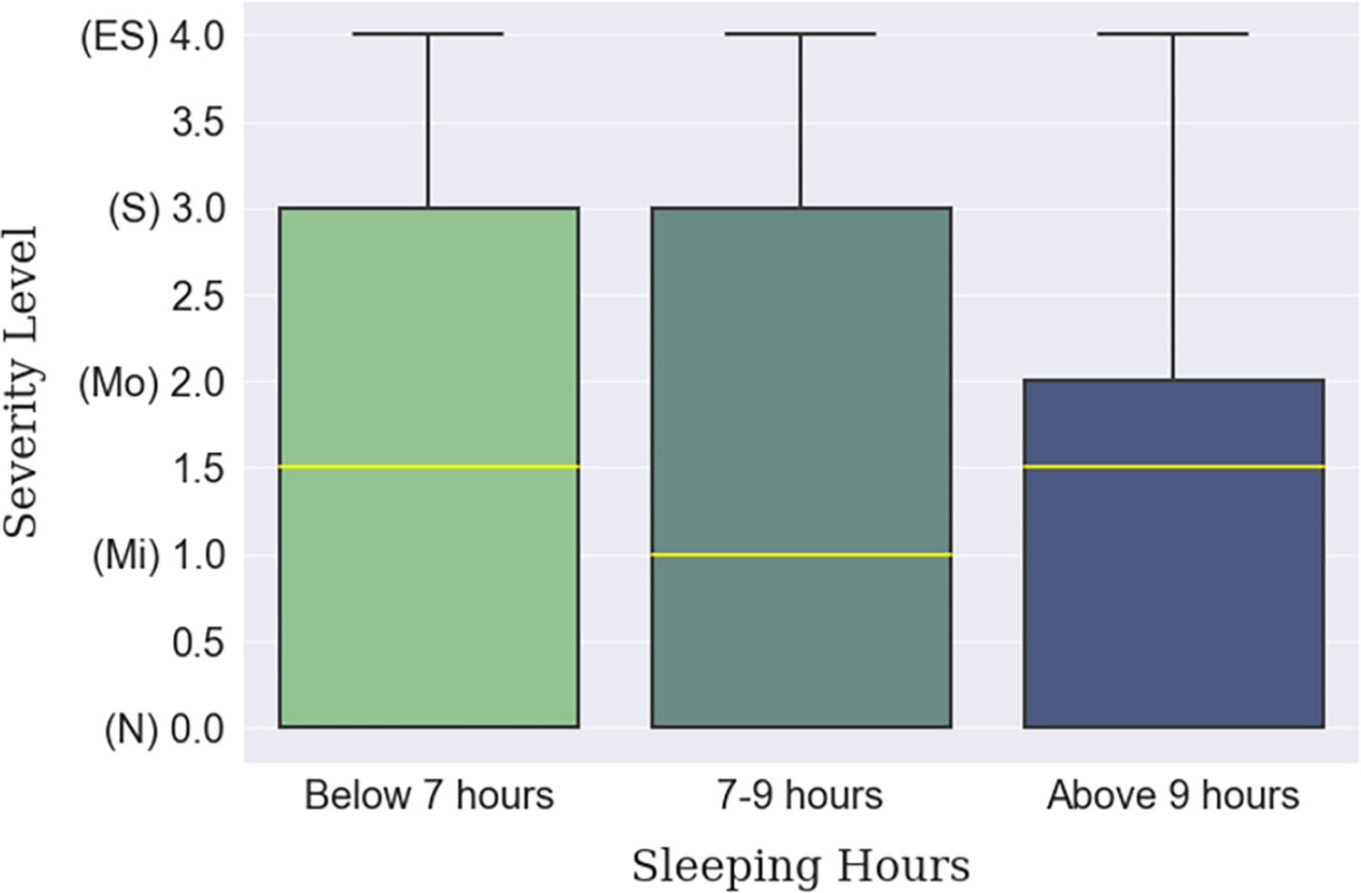
Sleeping hours-based depression severity distribution.

Depression was found to be more prevalent in big cities than in villages or small towns, which can be inferred from Table 12. Big city dwellers lived with a ‘Moderate’ median level of depression compared to the ‘Normal’ median level of the small town or village habitants, shown in Fig 8. This finding is backed up by internet connectivity status, empirically, which is more ubiquitous in the cities than in villages. Moreover, the respondents who were continuously connected to the internet were found to be slightly more depressed (Fig 9). The individuals who were suffering from lack of sleep were more depressed than those who were satisfied with the quality of their sleep. In fact, a gradual decline in depression can be seen among the respondents as their sleep satisfaction increased, as arrayed in Fig 10. Finally, those whose sleep duration was between 7–9 hours, were found to be experiencing less depression (median) than those who were oversleeping (more than 9 hours) or under sleeping (less than 7 hours), as shown in Fig 11.

**Table 12:** Result of the data analysis based on residence types.

Depression Severity Level	Village / Rural Area		Small City / Town		Big City	
	Count, N = 14	DASS-Depression Score (Mean ± Deviation)	Count, N = 98	DASS-Depression Score (Mean ± Deviation)	Count, N = 332	DASS-Depression Score (Mean ± Deviation)
Normal (N)	8	0.75 ± 2.12	50	2.2 ± 2.56	137	2.49 ± 2.84
Mild (Mi)	1	12 ± 0	13	11 ± 0.38	26	10.69 ± 2.19
Moderate (Mo)	3	16.67 ± 2.31	11	15 ± 0.81	57	16.21 ± 2.19
Severe (S)	1	24 ± 0	8	23.25 ± 1.83	43	23.9 ± 1.57
Extremely Severe (ES)	1	28 ± 0	16	34.5 ± 5.72	69	34.55 ± 5.29

#### 3.1.4 Statistical summary

**Fig 12:**
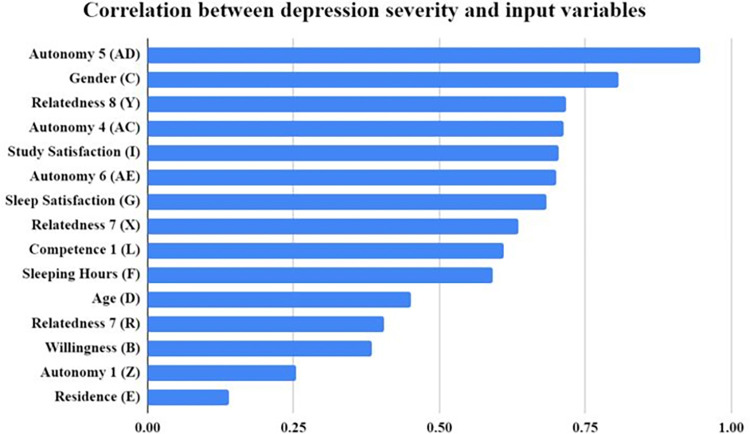
Comparison of accuracy using All features by performing (Chi-squared test).

The overall summary of the statistical data analysis is represented in Fig 12. The correlation between depression severity and the input variables is shown in terms of attribute importance by performing the Chi-squared test. The letters between the parenthesis designate the column in the dataset used in this study. Gender played a key role as a demographic variable when it comes to calculating depression severity scores. Age and residence are also seen to be affecting the severity score. However, the questionnaire items were found to be more crucial when determining a person’s depression severity.

### 3.2 Automated detection performance of the ML models

**Fig 13:**
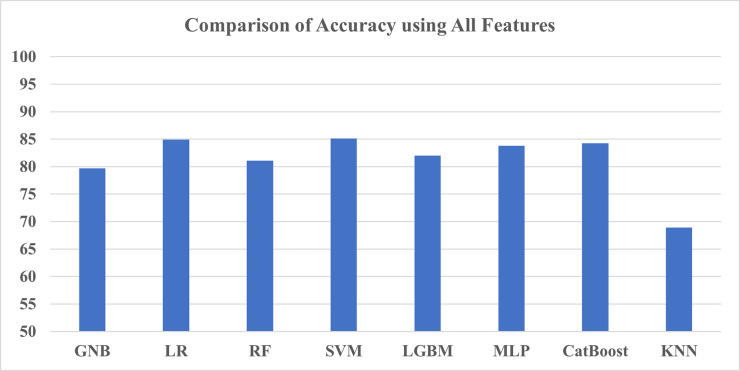
Comparison of accuracy using all features.

**Fig 14:**
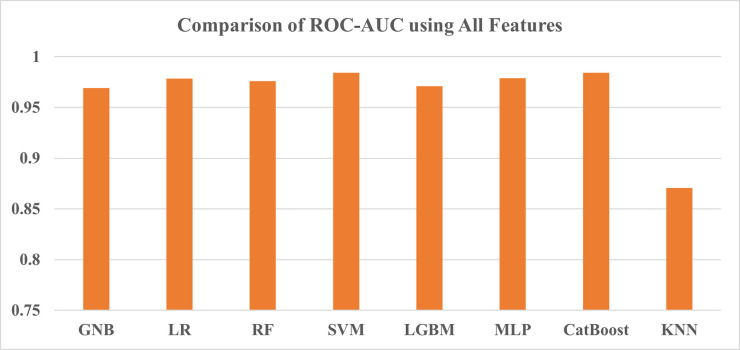
Comparison of ROC-AUC using all features.

For the first test, we have taken all features to train the models, and based on the findings shown in Table 13, SVM achieved the highest accuracy of 85.74% and F1-score of 0.8492. However, the CatBoost ROC-AUC score (0.9846) before hyperparameter tuning was the highest among other models’ AUC scores. The worst performing classifier was KNN, achieving the lowest score of all three metrics. Other than that, the accuracy values are largely close to each other along with the ROC-AUC score which can be seen in Figs 13 and 14 respectively.

**Table 13:** ML models performance using all features.

		Accuracy (%)		F1-Score		ROC-AUC	
**Algorithm**		initial	tuned	initial	Tuned	initial	tuned
1.	GNB	65.10	79.72	.6558	.7995	.9467	.9690
2.	LR	82.90	84.91	.8160	.8460	.9704	.9786
3.	RF	83.34	81.07	.8107	.7748	.9779	.9760
4.	SVM	75.91	**85.14**	.7426	**.8492**	.9158	.9841
5.	LGBM	81.77	81.99	.8103	.8080	.9666	.9711
6.	MLP	81.30	83.78	.8050	.8246	.9709	.9789
7.	CatBoost	84.24	84.25	.8335	.8220	**.9846**	.9843
8.	KNN	68.03	68.92	.6432	.6687	.8997	.8705

### 3.3 ML models with feature engineering results

To achieve a better performance out of the ML models and improve the automated detection process, different feature engineering methods were incorporated as discussed in the methodology. The comparative outcome among different features of engineering algorithms is presented next.

#### 3.3.1 Comparison between Chi-square test and RFE

**Fig 15:**
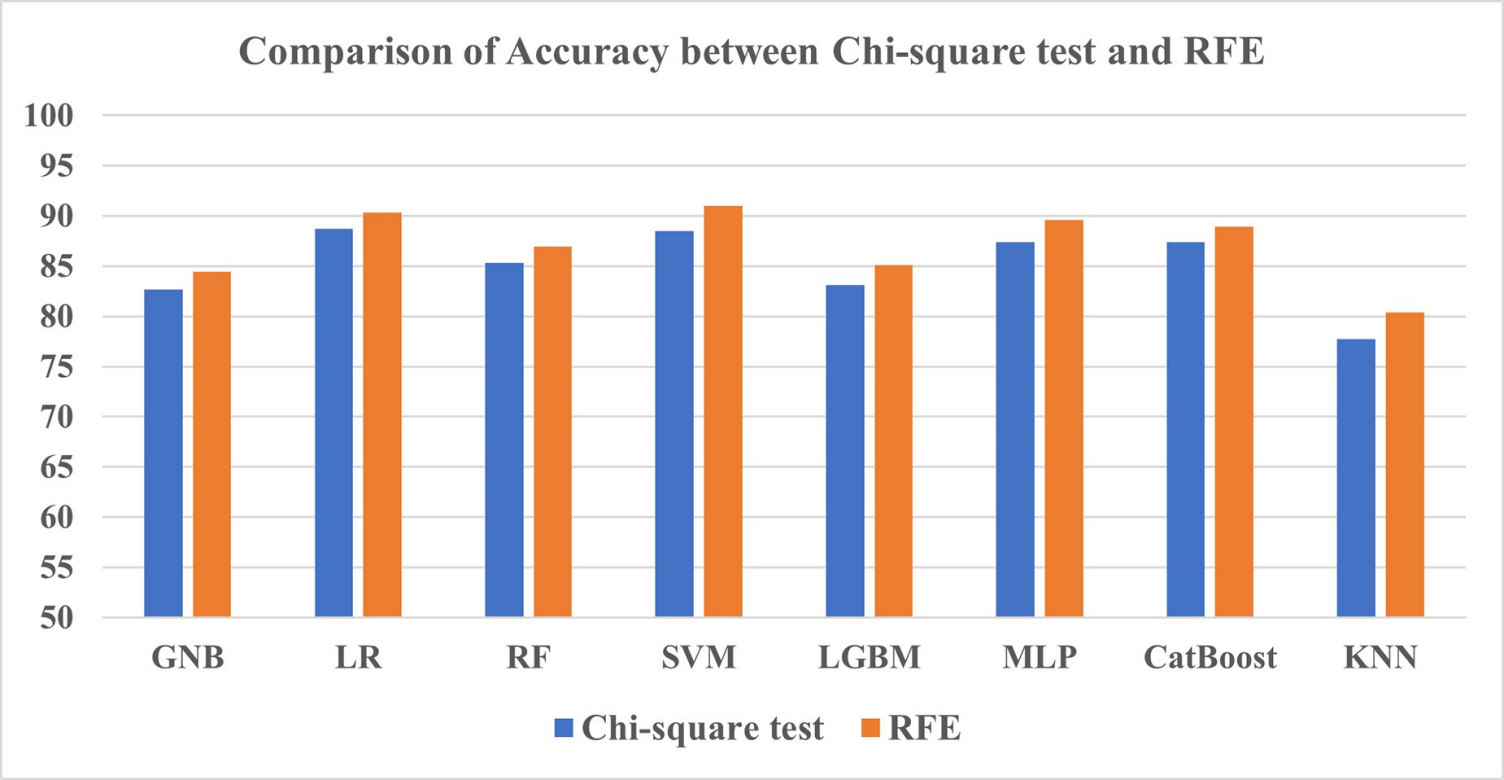
Comparison of accuracy between Chi-square test and RFE.

**Fig 16:**
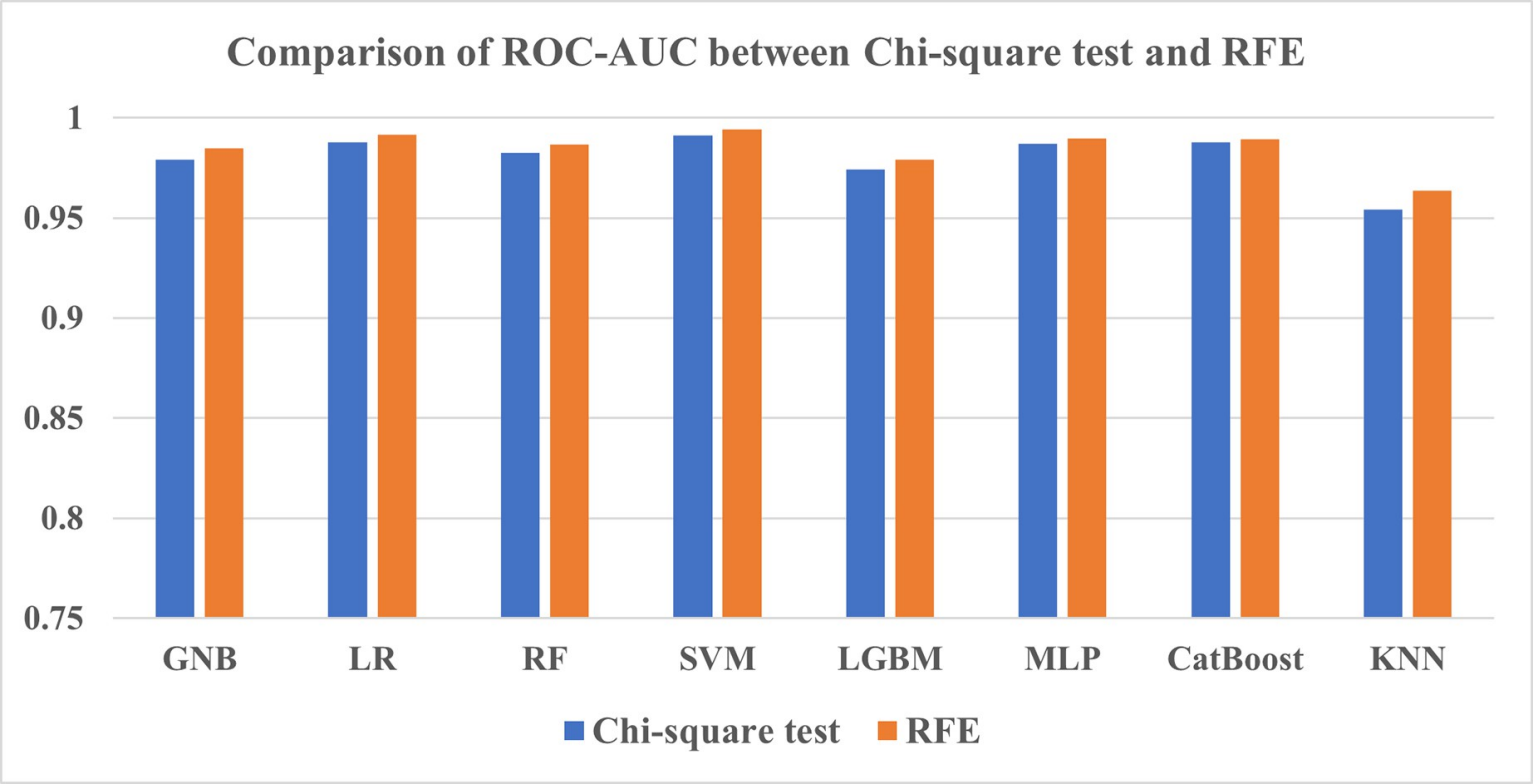
Comparison of ROC-AUC between Chi-square test and RFE.

From the feature selection results in Tables 14 and 15, SVM has achieved the highest score for both methods most of the time in terms of accuracy, F1-score, and AUC. A notable point here is that before tuning, SVM performed inadequately and came out in the last place among classifiers, which has been reversed after tuning. The only exception in which this classifier was outperformed by another classifier is in the Chi-square test by the LR model which achieved the highest accuracy of 88.74%. Having said that, KNN once again exhibited the lowest scores among all three metrics for both methods. Overall, the RFE method comparatively better assisted the ML models than the Chi-square test in this study’s findings as shown in Figs 15 and 16.

**Table 14:** ML models performance (Chi-square test).

		Accuracy		F1-Score		ROC-AUC	
Algorithm		initial	tuned	initial	tuned	initial	tuned
1.	GNB	78.83	82.66	.8044	.8329	.9737	.9792
2.	LR	82.88	**88.74**	.8074	.8821	.9767	.9877
3.	RF	84.24	85.36	.8236	.8364	.9819	.9826
4.	SVM	75.90	88.51	.7301	**.8837**	.9365	**.9912**
5.	LGBM	84.68	83.11	.8405	.8253	.9794	.9742
6.	MLP	80.30	87.38	.8108	8691	.9732	.9872
7.	CatBoost	84.69	87.39	.8389	.8664	.9862	.9878
8.	KNN	77.94	77.77	.7644	.7608	.9600	.9541

**Table 15:** ML models performance (RFE).

		Accuracy		F1-Score		ROC-AUC	
Algorithm		initial	tuned	initial	tuned	initial	Tuned
1.	GNB	81.30	84.46	.8261	.8427	.9775	.9847
2.	LR	84.46	90.31	.8248	.8990	.9822	.9915
3.	RF	87.61	86.94	.8657	.8575	.9869	.9867
4.	SVM	77.70	**90.98**	.7502	**.9079**	.9471	**.9942**
5.	LGBM	84.02	85.14	.83.43	.8449	.9787	.9790
6.	MLP	84.68	89.63	.8389	.8919	.9822	.9896
7.	CatBoost	88.51	88.97	.8797	.8833	.9891	.9895
8.	KNN	79.96	80.41	.7740	.7764	.9679	.9636

#### 3.3.2 Comparison between PCA and SparsePCA

**Fig 17:**
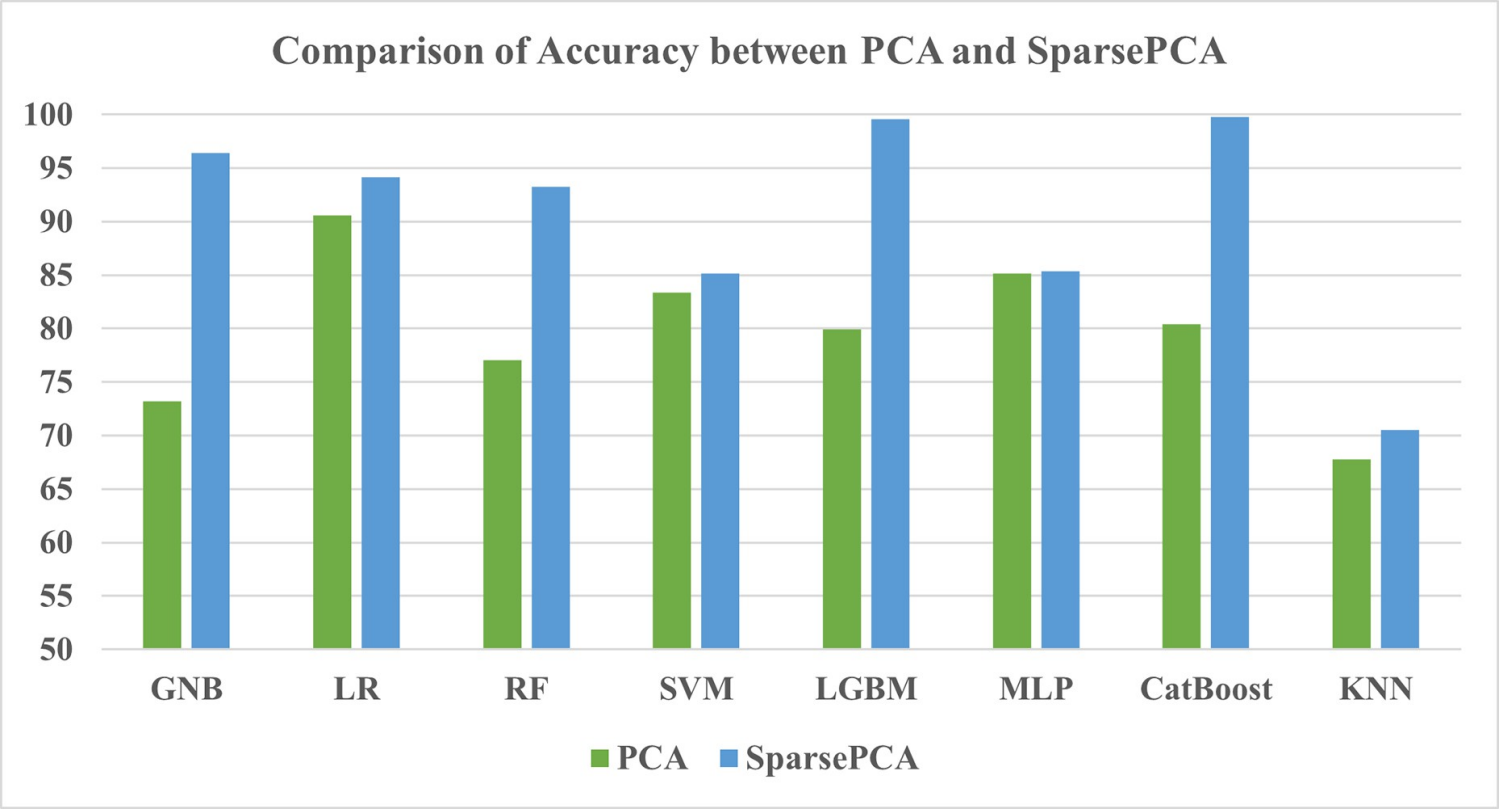
Comparison of accuracy between PCA and SparsePCA.

**Fig 18:**
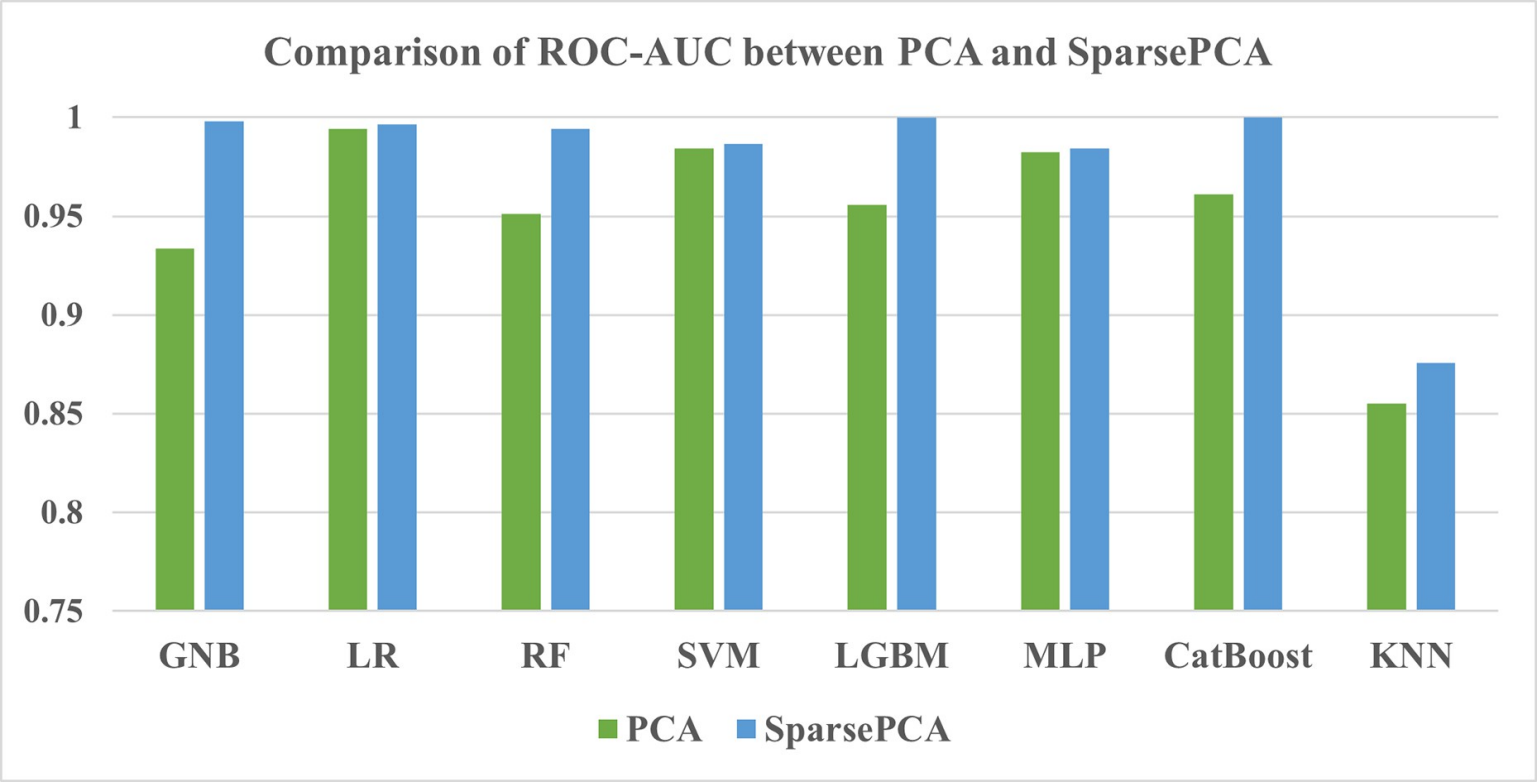
Comparison of ROC-AUC between PCA and SparsePCA.

When the ML models were trained with the feature extraction PCA method, the LR model consistently performed better than other models by achieving 90.55%, 90.35%, and 0.9943 for accuracy, F1-score, and ROC-AUC score respectively (Tables 16 and 17). Nevertheless, the SparsePCA method experienced a different model, the CatBoost, to overperform and become the best performing model among all, which was followed by LGBM with a marginal gap. Lastly, KNN again became the most subdued performing classifier for both feature extraction methods. Hence, it can be concluded that SparsePCA showed better performance than the PCA method, and the performance comparison is also depicted in Figs 17 and 18.

**Table 16:** ML models performance (PCA).

		Accuracy (%)		F1-Score		ROC-AUC	
Algorithm		initial	tuned	initial	tuned	initial	tuned
1.	GNB	70.50	73.19	0.6835	0.7033	0.9234	0.9337
2.	LR	82.21	**90.55**	0.7993	**0.9035**	0.9761	**0.9943**
3.	RF	75.45	77.02	0.7088	0.7287	0.9471	0.9513
4.	SVM	76.81	83.34	0.7381	0.8261	0.9461	0.9842
5.	LGBM	79.96	79.96	0.7872	0.7895	0.9584	0.9557
6.	MLP	78.83	85.13	0.7756	0.8354	0.9739	0.9823
7.	CatBoost	81.52	80.40	0.7976	0.7881	0.9667	0.9612
8.	KNN	69.83	67.79	0.6701	0.6570	0.9167	0.8551

**Table 17:** ML models performance (SparsePCA).

		Accuracy (%)		F1-Score		ROC-AUC	
Algorithm		initial	tuned	initial	tuned	initial	tuned
1.	GNB	95.50	96.39	0.9543	0.9634	0.9979	0.9982
2.	LR	85.14	94.15	0.8353	0.9411	0.9801	0.9966
3.	RF	92.12	93.23	0.9187	0.9289	0.9949	0.9943
4.	SVM	76.36	85.15	0.7291	0.8507	0.9502	0.9867
5.	LGBM	98.88	99.55	0.9884	0.9955	0.9999	0.9999
6.	MLP	81.74	85.36	0.8026	0.8402	0.9735	0.9844
7.	CatBoost	99.78	**99.78**	0.9977	**0.9977**	1	**1**
8.	KNN	72.97	70.50	0.7125	0.6876	0.9150	0.8758

In addition to the chart comparison, the mean value of the confusion matrices generated from all the tuned ML models when incorporating the SparsePCA feature extraction method have been shown in Tables 18–20.

**Table 18:** The mean value of confusion matrix for GNB, LR, RF (SparsePCA).

	Predicted Category	GNB					LR					RF				
		0	1	2	3	4	0	1	2	3	4	0	1	2	3	4
Actual Category	0	38.6	0.4	0	0	0	38.6	0.4	0	0	0	39	0	0	0	0
	1	0.6	6.8	0.6	0	0	0.6	7	0.4	0	0	0.2	5.6	2.2	0.2	0
	2	0	0.2	13.6	0.4	0	0	0.8	12.2	1.2	0	0	0	13.6	0.6	0
	3	0	0	0	9.6	0.8	0	0	1	9	0.4	0	0	2.2	7.4	0.8
	4	0	0	0	0.2	17	0	0	0	0.4	16.8	0	0	0	0	17.2

**Table 19:** The mean value of confusion matrix for SVM, LGBM, MLP (SparsePCA).

	Predicted Category	SVM					LGBM					MLP				
		0	1	2	3	4	0	1	2	3	4	0	1	2	3	4
Actual Category	0	38	0.8	0.2	0	0	39	0	0	0	0	38.4	0.6	0	0	0
	1	1.6	5.8	1.8	0	0	0	7.8	0.2	0	0	3.8	3.4	1.8	0	0
	2	0.2	1.2	11	2.2	0	0	0.2	14	0	0	0.2	1.2	14	0.8	0
	3	0	0	1	7.8	1.4	0	0	0	10.4	0	0	0	0.8	6.4	5.2
	4	0	0	0	3	14.2	0	0	0	0	17.2	0	0	0.2	1.4	15.6

**Table 20:** The mean value of confusion matrix for Catboost, KNN (SparsePCA).

	Predicted Category	CatBoost					KNN				
		0	1	2	3	4	0	1	2	3	4
Actual Category	0	39	0	0	0	0	37.2	1	0.8	0	0
	1	0	8	0	0	0	4.6	2.2	1.2	0	0
	2	0	0	14.2	0	0	4.4	2.2	7	0.6	0
	3	0	0	0	10.2	0	1.2	0.8	3.4	3.2	1.8
	4	0	0	0	0	17.2	0.2	0.2	0.6	3.2	13

## 4 Discussion

### 4.1 State of depression among the study groups

In this study, a machine learning approach was employed to detect students’ depression due to their continual connectivity with mobile devices and the Internet. A multi-class classification was performed on five different severity levels using the DASS-Depression scale. It was identified that the male students were comparatively less affected than their female counterparts, a finding consistent with the results of previous studies [63, 94]. The younger students were substantially more vulnerable to a higher degree of depression, classified as severe than were other students. In terms of ethnicity, it is hard to conclude which student group was more prone to experiencing issues associated with depression due to the different sample sizes of each ethnic group.

Previous studies revealed that students were more prone to be diagnosed with depressive symptoms from moderate to extremely severe levels compared to other cohorts of people [95]. This situation became worse after the appearance of the COVID-19 epidemic where mild to extremely severe level depression was observed among 46% of the students [96]. Specifically, among university students, the percentage was raised to 72% [97]. Students who thought themselves to be inferior performers in online education were more likely to be depressed more than the others [98]. In this study, considering the impact of continual mobile connectivity, less than half of the students (43.4%) were found to be at the mild-to-extremely-severe level of depression. This can serve as positive feedback for the hybrid mode of learning as previous studies reported higher percentages [97]. However, this might also be partially explained by another study which revealed that about 32% of the students were facing depression symptoms after reopening the campus [99]. However, this study also shows that satisfaction with academic activities was highly correlated with the students’ depression severity level.

In addition, sleep satisfaction and internet connectivity were found to be crucial factors for depression prevalence among the students [98, 100]. In this study, participants with low sleep satisfaction were found to be in higher depression severity classes, and consistent internet connectivity was found to have a further influence on the depression severity level.

It is worth mentioning that a significant portion of the students observed in this study had none to very minimal depression levels. For example, 44% of the data belonged to Group 0 whose students were normal in terms of their depression level. However, more than half of the students may have experienced mild to severe level depression at some point in their lives, for example, 19% of students had extremely severe (group 4), 12% had severe (group 3),16% had moderate (group 2), and 9% had mild (group 1) depression level.

It is worth mentioning that the data was not balanced in the current study as per the standard practice. This study was conducted considering real-life applications where the balanced student distribution in depression levels was rarely observed [101]. As a result, the number of students having no (or mild) stress might be larger than the number of people having high stress, or vice versa. For instance, 44% of the participants had no depression issues, which was the greatest among all the categories under consideration. A similar pattern of data variability is observed in the literature which held the same assumption, i.e., most people around the world possess none to mild depression issues [102–104]. It has been observed that, in this study, the adoption of data variability in different depression levels does not have any significant impact on the accuracy level of the ML algorithms.

### 4.2 Best performing ML pipeline

When analyzing the performance of the algorithms, it can be said that the metrics showed quite high value for most of the algorithms. Therefore, if the attribute number gets higher or the feature-set becomes more non-linear in nature, the prediction of depressive disorder in multiple classes (mild, moderate, severe, extremely severe) can be achieved with relatively high accuracy. Furthermore, tuning the hyperparameters enhanced the results in most of the cases. One notable characteristic of the algorithm is the SVM’s popularity and use in clinical applications [105]. Most of the research work has also employed SVM which can lead to oversight of the new and updated algorithms’ implementation and performance analysis.

The research studies from Bangladesh, which have been laid out chronologically in **Table 21**, mostly performed a binary classification of identifying whether a person is depressed or not. In this study, the multi-class classification performance is like the binary classification of other studies [106, 107]. The performance of the KNN algorithm was lower than other algorithms in several studies as well as in this study, which can be regarded as KNN being not able to fit the data properly in the depression studies [108, 109]. Other than that, traditional classifiers have been satisfactory in terms of exhibiting higher performance.

**Table 21:** Comparison with other research on automated depression detection in Bangladesh.

Ref.	Respondent number	Questionnaire used (excluding demographic measures)	Feature Engineering	Algorithms employed	Results (best performed model)
[50]	935	Proprietary 16-item questionnaire, Beck Depression Inventory-II (BDI-II), DASS 21 (Bangla Version)	Twenty selected (procedure not mentioned)	KNN, RF, SVM	RF acc: 75% f1-score: 0.60 roc-auc: 0.797
[107]	461	PROMIS Emotional Distress-Depression Short Scale, Internet Addiction Test (IAT), Rosenberg Self-esteem Scale (RSE)	Chi-square, mRMR	LR, NB, RF, C4.5 Decision Tree (DT), and KNN	LR acc: 86% roc-auc:0.933
[108]	not mentioned	30-item depression scale, 35-item anxiety scale (developed by native researchers)	not mentioned	Linear Regression, KNN, SVM, LDA, Convolutional Neural Network (CNN)	CNN acc: 96.8%
[109]	765	Proprietary depression risk factor-related questionnaire	not mentioned	’SAMME.R’ AdaBoost, KNN, RF, DT	AdaBoost acc: 98% f1-score: 0.981%
[61]	520	Career and Job Seeking stress-related 10 questions, Patient Health Questionnaire (PHQ-9)	Chi-square, Backward elimination method	LR, Gradient Boosting (GB), Linear SVC, RF, Multinomial NB, Stacking classifier	Stacking Classifier acc: 77.45% roc-auc: 0.80
[110]	800	Proprietary 29-item depression questionnaire	not mentioned	LR, Simple LR, SMO, LMT, IBK, J48, K-star, Classification Via Regression (CVR)	SMO acc: 98.94%
[63]	604	Proprietary 30-item questionnaire, Burns Depression Checklist (BDC)	SelectKBest, Minimum-Redundancy-Maximum-Relevance (mRMR), and Boruta algorithm	KNN, AdaBoost, GB, XGBoost, Bagging classifier, Weighted voting classifier	AdaBoost acc: 92.56% f1-score: 0.9379 roc-auc:0.96
[106]	410	Proprietary job-related questionnaire	not mentioned	RF, RF Regressor, NB, KNN	RF acc: 99.48%
[62]	2121	PHQ‑9, Generalized Anxiety Disorder Assessment (GAD‑7)	not mentioned	LR, RF, SVM, Linear Discrimination Analysis (LDA), KNN, NB	SVM acc: 91.49%
This study	444	Digital stress and competency related questionnaire, DASS-Depression	Chi-square and RFE (Feature selection), PCA and SparsePCA (Feature extraction),	GNB, LR, RF, SVM, LGBM, MLP, CatBoost, KNN	CatBoost acc: 99.78 f1-score: 99.77 roc-auc: 1

### 4.3 Effect of feature engineering on ML models

Few studies from Bangladesh have paid attention to the feature engineering step, which benefits the automated detection pipeline. The current study and the one by Zulfiker et al. [63] both found the boosting algorithms’ performance to be higher than traditional ML classifiers by incorporating this step. The accuracy rose by about 3% with the Chi-square test feature selection method and about 5% with the RFE method. Meanwhile, the PCA saw a 5% increase in accuracy and for SparcePCA, a significant jump of 15% could be observed from the standard performance of the ML algorithms. This finding needs to be further investigated in future studies so that more clinical appliances benefit from feature engineering methods. However, there is less variation in the selection of the feature engineering methods, which also needs to be broadened in future studies. The following **Table 21** summarizes the comparison of the current study with other research studies on automated depression detection in Bangladesh.

### 4.4 Implications of the study

This study is intended to make a three-fold research contribution. First, student digital well-being and level of depression stemming from the mobile connectivity is illustrated based on different variables (e.g., age, gender, geographic location, academic year of study, sleeping habits, etc.) that provide new insights into how these factors are related to students’ depression.

Second, an automated model of depression detection and classification process is explored by employing eight ML algorithms namely Gaussian Naïve Bayes, Logistic Regression, Random Forest, Support Vector Machine, Light Gradient Boosted Machine, Multi-layer Perceptron, CatBoost, and K-Nearest Neighbor. The automated detection is examined by incorporating feature selection (Chi-square test and Recursive Feature Elimination) and feature extraction (Principal Component Analysis and SparsePCA) methods to find out the best performing ML pipeline. This provides direction for the researcher to consider the most suitable model for the detection of student depression in future studies.

Finally, this study is intended to serve as a valuable insight into the body of depression literature and for the policymakers, teachers, educators, and medical practitioners globally in different disciplines and domains. The global pandemic has not only disrupted the physical delivery of education, but it also affected students’ mental health. In this regard, this study provides new insights into how the students’ mental health is affected by the overuse of mobile devices and internet connectivity.

### 4.5 Limitations of the study

There are a few limitations to this study. Since this study constitutes data primarily from Bangladesh along with other OIC countries, the inhabitants from these regions are the most represented in the results. The respondents only consisted of students in this study. The sample could be more diversified so that other classes and professions of society could be studied. The self-reported survey data is subject to produce a misinterpretation of the data with possible biases. Also, this study is investigative in nature and no experimental design was employed to compare study findings with a set of clinical samples.

The sample size is moderate for this domain since the difficulty of acquiring mental health-related data is quite high. In particular, higher age groups and overseas respondents contributed a small portion to the survey data which suffers from being versatile and may not be truly representative for all the participants.

On the technical side, the class imbalance issue as well as the over-performance of some classifiers were also evident when the ML model has been trained with the SparsePCA method. Though the results were mostly representative of all five classes, there was some imbalance in the dataset. This can affect the outcome of the study and make the results more applicable to those classes that have a higher percentage of students. Also, data balancing techniques have not been employed in this study. We intend to make a comparative analysis between the balanced and imbalanced nature of this dataset in future work.

Methodologically, this is an investigative study and no interpretability methods like SHAP or LIME have been employed in this study. The interpretability methods may have different findings and interpretations that can shift the focus of the study in a different direction. Lastly, the study did not utilize any modality such as video or images other than textual responses and thus the findings may be less comprehensive in nature.

## 5 Conclusion

Mental health is becoming of increasing concern worldwide. In a South Asian country such as Bangladesh, the importance of mental health-related disorders is even more since this country is plagued with so many different confounding issues. As a result, the factors that play a crucial role in instigating depression cannot be circumscribed with statistical analysis alone. Furthermore, online or hybrid modes of learning have a higher probability of becoming more accepted in the future and thus further accelerate the adoption of continual mobile connectivity among educators and students engaged in teaching and learning. Thus, in anticipation of a substantial increase in mental health issues in response to the new normal mode of delivery, it seems timely for a smart healthcare system to be alert to the need to provide greater support to depressed individuals.

In this research, the potential vulnerability of student groups to depression was assessed through an online survey employing different ML models with multiple feature engineering methods to extract the best-automated depression detection pipeline. Around 44% of the respondents were found to be rated “normal” whereas a total of 47% of respondents were found to be rated at the moderate-to-extremely severe depression level. Besides, data analysis also revealed some insights about depression among the students based on different age groups and ethnicity.

The performance analysis of the ML models reveals that when feature engineering methods like the Chi-square test and RFE are added, the accuracy metric increases from 3% to 5%. In a similar vein, PCA and SparsePCA methods increased the accuracy level from 5% to 15%. The SparsePCA method of feature extraction combined with the CatBoost classifier performed the best. Moreover, a comparison with other research works has been demonstrated to estimate the overall condition and the factors that are responsible for causing depression. In future, a larger survey may be conducted with varying cultural and societal differences to reveal a broader picture of students’ levels of depression. Besides, neural networks need to be examined to achieve a better understanding of the automated psychiatric consultancy process.

## Supporting information

S1 Dataset (CSV)
